# Horizontal Gene Transfers from Bacteria to* Entamoeba* Complex: A Strategy for Dating Events along Species Divergence

**DOI:** 10.1155/2016/3241027

**Published:** 2016-04-27

**Authors:** Miguel Romero, R. Cerritos, Cecilia Ximenez

**Affiliations:** Faculty of Experimental Medicine, Experimental Immunology Laboratory, National Autonomous University of Mexico, Dr. Balmis 148, Colonia Doctores, 06720 Mexico City, Mexico

## Abstract

Horizontal gene transfer has proved to be relevant in eukaryotic evolution, as it has been found more often than expected and related to adaptation to certain niches. A relatively large list of laterally transferred genes has been proposed and evaluated for the parasite* Entamoeba histolytica*. The goals of this work were to elucidate the importance of lateral gene transfer along the evolutionary history of some members of the genus* Entamoeba*, through identifying donor groups and estimating the divergence time of some of these events. In order to estimate the divergence time of some of the horizontal gene transfer events, the dating of some* Entamoeba* species was necessary, following an indirect dating strategy based on the fossil record of plausible hosts. The divergence between* E. histolytica* and* E. nuttallii* probably occurred 5.93 million years ago (Mya); this lineage diverged from* E. dispar* 9.97 Mya, while the ancestor of the latter separated from* E. invadens* 68.18 Mya. We estimated times for 22 transferences; the most recent occurred 31.45 Mya and the oldest 253.59 Mya. Indeed, the acquisition of genes through lateral transfer may have triggered a period of adaptive radiation, thus playing a major role in the evolution of the* Entamoeba* genus.

## 1. Introduction

*Entamoeba* genus is formed by morphologically similar amoebas; most of them are intestinal parasites that can infect several hosts [1].* Entamoeba histolytica* is one of the most important intestinal protozoan parasites in humans causing amoebic colitis; they can also invade the liver causing amoebic liver abscess. It is estimated that this parasite causes 70,000 deaths worldwide each year [2]. Furthermore, the* E. dispar* species is morphologically almost identical to* E. histolytica*. However, until today, this has been considered as a commensal organism of the human gut. Nevertheless,* E. dispar* has been detected in patients with symptomatic amoebic colitis and also in the material of amoebic liver abscesses [3]. Very recently, a novel lineage of the* Entamoeba* genus has been detected in the intestine of rhesus macaques* Macaca mulatta*. Moreover, it has been proposed as a candidate to revive the name* E. nuttallii* for this lineage, particularly due to its genetic characteristics.

*E. nuttallii* infects captive and wild macaques and is capable of causing abscesses in hamster's livers [4]. The species of* Entamoeba invadens* infects reptiles and causes colitis, liver abscesses, and, sometimes, acute death. It has been used as the main encystation model for* Entamoeba* species, since the in vitro culture of* E. dispar* can excyst producing the trophozoites and, thereafter, these trophozoites can undergo encystation in vitro. Phylogenetic reconstructions performed by Stensvold et al., in 2011, based on sequences of the gene for the small subunit of rRNA, clustered together* E. histolytica* and* E. nuttallii* and, basal to the latter node, branched those from* E. dispar*. SSURNA sequences from* E. invadens* branched together with those of* E. ranarum*. Both sequences formed the sister group of a node consisting of more than two-thirds of the* Entamoeba* species included in the analysis [1].

It is well known that horizontal gene transfer (HGT, or lateral gene transfer, LGT), of genetic material between unrelated individuals, has played a significant role in prokaryotic gene acquisition and genome evolution [5, 6]. Over the past few years, its importance in eukaryotic evolution has been reevaluated as it has been found in a higher frequency than expected and related with adaptation to certain niches [7]. Despite its presence in multicellular organisms such as Bdelloid rotifers [8], it is more likely to occur in unicellular eukaryotes [9]. Alsmark et al., in 2013, analyzing several genomes of protozoa found that* Leishmania mayor*,* Entamoeba histolytica*, and* Trypanosoma brucei* have the major percentage of genes acquired by lateral transfer with 0.96, 0.68, and 0.47, respectively [10]. Although the phylogenetic discrepancy has been the most reliable method to identify horizontally transferred genes, this latter procedure has been criticized, due to the following arguments: it is known that it might be modified due to methodological artifacts such as substitution saturation or long-branch attraction. Because there are only four bases that constitute nucleic acids, there is a relatively high probability that two nucleotide sequences might share the same bases in a random site by mere chance. This phenomenon is caused regularly by the high molecular substitution rate present in the locus, and its particular unwanted results are the loss of phylogenetic information and the possible high similarity between unrelated sequences. As a whole, this phenomenon is known as substitution saturation and is one of the main problems when analyzing molecular data [11]. HGT may be inferred amiss due to substitution saturation and it must be taken into account on every phylogenetic analysis.

The divergence time estimation for protozoan species is commonly a challenging endeavor, especially at the node calibration step. Even though some protozoan taxa might have fossil record, the most common strategy to calibrate date estimates is the indirect calibration based on animal or plant fossils with a specific underlying biological hypothesis [12]. In fact, the calibration of time estimates performed with protozoan fossil record has proven to be unpractical for extant taxa [13]. Although it has been suggested from gene comparisons that the divergence time between* E. histolytica* and* E. dispar* may have occurred some tenths of millions of years ago [14–16], until now no exhaustive research has been performed on the subject.

In the first annotation of the genome of* E. histolytica* HM1:IMSS reported by Loftus et al., a list of 96 HGT candidates was included, many from bacterial donors [17]. Later, in 2007, Clark et al. updated the analyses and sorted these 96 candidates into different categories according to their consistency in Bayesian and maximum likelihood distance bootstrap trees [18]. From the 96 original candidates, 41 remained strongly supported; 27 turned to be more weakly supported than before; the lateral gene transfer hypothesis of 14 candidates was weakened by increased taxonomic sampling; 9 candidates were found in other microbial eukaryotes; and, in the remaining 5 cases, vertical gene transfer is now the simplest explanation for the observed topology. Horizontal gene transfer remains the strongest hypothesis to explain 68 of the 96 original topologies [18]. But the number of LGT candidates can change according to phylogenetic methodology. For example, recently, in a study by Grant and Katz, in 2014, it is concluded that there are 116 genes of* Entamoeba* having a bacterial or archaeal origin [19]. In laboratory, Field et al. showed that the acetyl-CoA synthetase and the adh1 genes of* E. histolytica* share a common evolutionary history, more related to prokaryotes than other eukaryotes, and suggested that these genes were transferred early [20]. Hand in hand, Nixon et al. tried to demonstrate that genes for the anaerobic metabolism in* Giardia* and* Entamoeba* genera were obtained laterally; while there was no enough data available to achieve that goal, the authors did reject the amitochondriate fossil and the hydrogen hypotheses to explain the resemblance of these genes to prokaryotic sequences [21].

The objective of this study is, primarily, to estimate the divergence time between* E. histolytica*,* E. dispar*, and* E. invadens* and then date HGT events of a representative genes, thereof, through the evolution of these species of* Entamoeba*. Representative gene was taken from the list of 68 candidates mentioned above and an additional analysis carried out to distinguish different levels of saturation rates in the DNA sequence, hypothesizing convergence or ancient HGT.

## 2. Methods

### 2.1. Gene Selection and Sequence Alignments

Accession numbers of the 68 well supported candidates were obtained from the list in Clark et al., 2007, and then searched in the Amoeba DB database, http://amoebadb.org/amoeba/ [22], in order to get the amino acid and coding sequences.

The following genes were selected to carry out the* Entamoeba* divergence time estimations: DNA-directed RNA polymerase I subunit RPA2 (EHI_186020), elongation factor 2 (EHI_189490), actin (EHI_131230), tubulin gamma chain (EHI_008240), and clathrin heavy chain (EHI_201510) since they are single-copy, housekeeping genes that were not obtained by HGT.

Each one of the HGT candidate amino acid sequences was used as a query against the NCBI Protein Reference Sequence Database (RefSeq) [23] with the BLASTp algorithm [24], using default parameters. The top 50 blast hits were collected for further analyses.

The homologous amino acid sequence from* E. nuttallii* was included in the analyses to calibrate the HGT divergence time estimates. Each* E. histolytica* HGT candidate was used as a query against the* E. nuttallii P19* open reading frame translation database with the BLASTp algorithm. Only the top hit was collected and discarded, if the query coverage and/or identity were less than 60%.

In order to estimate the divergence time of housekeeping genes, sequences from* E. dispar* were downloaded and then corresponding orthologs were searched against the open reading frame translation database of* E. histolytica*,* E. nuttallii*, and* E. invadens* (available at http://amoebadb.org/common/downloads/) using the BLASTp algorithm, with default parameters. In addition, homologs of the former were looked for in the amino acid sequence database from* Dictyostelium discoideum* (available at http://dictybase.org/) [25] also using a BLASTp search. Only the top hit was collected and discarded if the query coverage or identity was less than 60%.

Each amoeba sequence was aligned with its amoebic ortholog (when a report existed in the Amoeba DB database) and with the prokaryotic sequences found by the BLAST search. In every study, amino acid sequences were aligned using the program Clustal W [26] and then their codon sequences according to the amino acid alignment with Biopython scripting [27]. Sequence alignments were inspected manually and edited to remove synapomorphies and codons with sequencing errors.

### 2.2. Substitution Saturation Test

Distance matrices were built for the nucleotide alignments. The nucleotide substitution model used was the Maximum Composite Likelihood, with a gamma distribution for the rate variation among sites. All codon positions were included and ambiguous positions were removed for each sequence pair. This analysis was conducted in MEGA software [28].

For each evolutionary distance matrix, sequences with low distance values (equal to or less than 0.1 standard deviations) according to the* E. histolytica* sequences were selected to make a shorter sequence alignment, including only closely related sequences, according to the distance matrices.

Substitution saturation indexes Iss. and Iss.c [12, 29] were calculated for each alignment, considering the three positions of each codon. Whenever a sequence alignment showed substantial saturation after the first analysis, the indexes were calculated again, though we remove the third position of each codon to avoid the possible substitution saturation due to the degeneracy of the genetic code. In these assays, statistical significance value was set at *P* < 0.05. These tests were executed using the package DAMBE [30].

Tree topology examination was necessary to decide whether the alignment was phylogenetically informative for those alignments that showed substantial saturation when excluding the third position of each codon.

### 2.3. Phylogenetic Analyses

In order to evaluate the phylogenetic relevance of the shorter alignments that presented substantial saturation when removing the third position of each codon, the substitution saturation tests were introduced to the program MrBayes 3.2 [31]. The number of run MCMC generations was 500,000, excluding the third position of each codon; every 125 generations a tree was sampled. Whenever a tree from the latter resulted in asymmetrical topology, the HGT candidate was discarded from the analysis.

In all cases the GTR+I+G nucleotide substitution model was employed, and 25% of tree samples were discarded as burn-in. A consensus tree was constructed from the remaining samples, and then it was inspected manually and edited using Dendroscope [32].

Constructions of consensus trees for donor group designation were made using two different approaches: maximum likelihood and Bayesian phylogenetics. 61 sequence alignments were introduced to the program jModeltest2 [33], in order to find the sequence substitution model that best fitted the observed alignment. Eleven substitution schemes were used, along with relative frequencies per base, proportion of invariable sites, and the variation of substitution rates along the alignment. The base tree to perform each analysis was built with the BioNJ algorithm and the NNI search algorithm. Finally the AICc criterion was used to select the best model for each alignment.

Maximum likelihood trees were built for each alignment by the program PhyML 3 [34]. In each case, the base tree was built with BioNJ and the best tree whether from NNI or SPR search algorithms was selected. One hundred bootstrap tests were executed per alignment. The same strategy was included in the input for the PhyML software for the candidates that passed the saturation tests, when ignoring the third position of each codon.

Similarly, the alignments that presented no substitution saturation in the first saturation analysis were used as input to construct trees by the program MrBayes [31]. 1,000,000 MCMC generations were run sampling a tree every 200 generations. Also, 1,000,000 MCMC generations were run excluding the third position of each codon for candidates that presented no substitution saturation in the second saturation analysis; a tree was sampled every 200 generations. In both cases, the GTR+I+G substitution model was used, and a consensus tree was built after discarding 25% of the resulting topologies as burn-in. For each of the 61 candidates, the bootstrap values of the coincident nodes in the maximum likelihood trees were added manually to the resulting topology of the Bayesian phylogenetic analyses using the program Dendroscope (Supplementary Information, in Supplementary Material available online at http://dx.doi.org/10.1155/2016/3241027).

### 2.4. Bayesian Divergence Time Estimates

Two sets of estimations were performed: first,* Entamoeba* divergence time was calculated using a set of five housekeeping genes: DNA-directed RNA polymerase I subunit RPA2, elongation factor 2, actin, tubulin gamma chain, and clathrin heavy chain. Orthologs from* E. histolytica*,* E. nuttallii*,* E. dispar*,* E. invadens*, and* D. discoideum* were included in the dataset. The input tree used for the analysis was the following: ((((*E. nuttallii*,* E. histolytica*),* E. dispar*),* E. invadens*),* D. discoideum*).

Then, the HGT event dates were evaluated only with selected candidates, after considering three criteria: well supported branching in the Bayesian phylogenies, assigned donor group at least at the phylum level, and the presence of an ortholog in* E. nuttallii*. The dataset for each estimation included sequences contained in the alignments and used for the phylogenetic reconstructions from (i)* E. histolytica*, (ii)* E. dispar* and/or* E. invadens*, (iii) up to four randomly chosen sequences from the resulting sister group of* Entamoeba* cluster in the Bayesian phylogenies referred to as “a,” “b,” “c,” and “d,” (iv) up to three randomly chosen sequences from the resulting out-group in the Bayesian phylogenies referred to as “x,” “y,” and “z.” Moreover, the homologous sequence from* E. nuttallii* was aligned by-eye with the rest of the dataset. A common user-input tree would look like this: (((((*E. nuttallii*,* E. histolytica*),* E. dispar*),* E. invadens*), ((a, b),(c, d))), ((x, y), z)), even though the relationships within the sister group (a, b, c, and d) might vary.

The estimations were carried out using the programs Estbranches and Multidivtime [35, 36], following the step by step manual by Rutschmann [37]. The node between* E. nuttallii* and* E. histolytica* was used to calibrate the divergence estimations. Since* E. nuttallii* has only been isolated in rhesus macaques and* E. histolytica* has been found in feces from wild baboons (*Papio* sp.) [38, 39], we assumed that the* E. nuttallii* lineage diverged from* E. histolytica* at the same time that the primate lineages* Macaca* and* Papio* did. Paleontological evidence suggests that this divergence occurred after 8 Mya, but before 4 Mya [40, 41]. Consequently the node was calibrated between 5.5 and 6.5 Mya. For each alignment, the program Baseml [42] with the F84+G model was used to estimate nucleotide frequencies, transition/transversion rate ratio (parameter *κ*), and rate heterogeneity among sites (shape parameter *α*). Then, the maximum likelihood of the branch lengths of the tree and the variance-covariance matrix were estimated by the Estbranches program. Finally, a Bayes MCMC analysis was performed with the program Multidivtime, to approximate the posterior distributions of substitution rates and divergence times. A total of 5,100,000 generations were run, 100,000 were discarded as burn-in, and then a sample was taken every 100 generations.

For the* Entamoeba* divergence time, the five housekeeping genes were analyzed simultaneously, 5,100,000 generations were run, 100,000 were discarded as burn-in, and then a sample was taken every 100 generations. Time units were set to million years and referred to as “million years ago” (Mya). For the prior parameters, we selected 100 time units between the tip and the root of the tree, with a standard deviation of 50 time units, and an oldest time value of 300. For each candidate, the mean and standard deviation of prior distribution for the rate of molecular evolution at the in-group root node were set as the median of the evolution rates provided by Estbranches. The divergence time estimates were carried out in triplicate to confirm similar results of the analysis between repetitions. Results are showed as Mya ± the standard deviation provided by Multidivtime.

## 3. Results

### 3.1. Substitution Saturation Tests

Substitution saturation indexes Iss. and Iss.c [12, 28] were calculated for each alignment considering the first two or the three positions of each codon. The index Iss. is a measure of entropy of a given nucleotide sequence alignment. The index Iss.c is the measure of entropy of a simulated sequence alignment that shares the number of sequences and number of sites with the former but has a random distribution of nucleotide bases. Hence, if the Iss. value approaches that of Iss.c, it is a signal that the sequence alignment holds high substitution saturation. Both indexes were calculated for each shorter alignment. When using the three sites of each codon, 38 alignments displayed lower Iss. values than their respective Iss.c values; these differences were statistically significant (*P* < 0.05), implying little saturation. In 14 cases, the differences between Iss. and Iss.c were not statistically significant. Other 10 candidates display the same behavior, and in the remaining 6 cases the value of Iss. index was higher than Iss.c and, also, differences were statistically significant. In the second essay, in which the first 38 sequences were not included, every third position of each codon was ignored, in order to avoid the possible substitution saturation observed due to the degeneracy of the genetic code. Altogether, 15 alignments resulted in a significantly lower Iss. index, and other 4 alignments had a significantly higher Iss. than their respective Iss.c. The remaining 11 alignments showed nonsignificant differences; therefore tree topology was needed to evaluate their phylogenetic usefulness. To this end, 11 trees were built: 3 of them showed asymmetrical topology and 8 presented symmetrical topology. The respective HGT candidates from the 3 asymmetric trees, alongside those candidates from the 4 alignments whose Iss.c values were significantly higher in the second test, were permanently discarded from the research, since our results strongly denote that these alignments lack phylogenetic information.

### 3.2. Bayesian Phylogenetics and Putative Donor Groups

**Figure 1:**
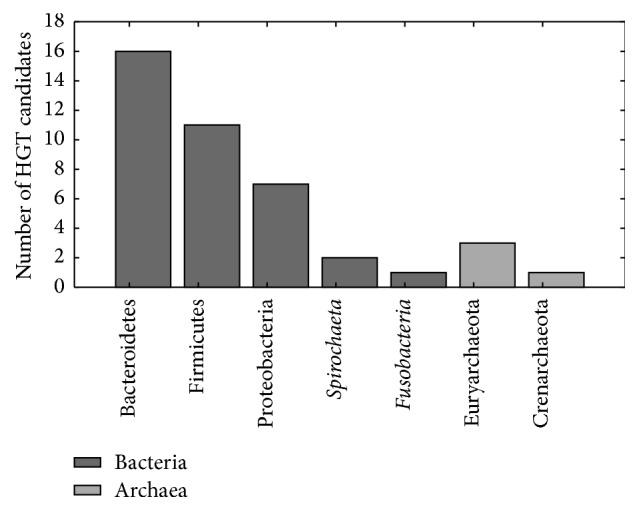
Number of genes obtained by each donor phylum identified in phylogenetic trees.

The assays were carried out with the lingering 61 candidates, using the complete coding sequence alignments. Phylogenetic analyses were made with the program MrBayes [31], 1,000,000 MCMC generations were run sampling a tree every 200 generations, using the first two or the three positions of each codon. When evaluating donor groups in the 38 trees constructed with complete codons, it was possible to locate a donor group at least at phylum level (Figure 1), as well as in 15 trees built, excluding the third position.

On the other hand, in three different cases, it was only possible to assign a donor group at the level of domain because the sister group of the* Entamoeba* cluster was formed by sequences that belonged to different phyla but from the same domain. A total of 61 consensus trees were built (Supplementary Information). Donor taxa could not be identified in the remaining five topologies, due to the different domains included and no apparent association with the amoebic genes. The domain Archaea was assigned to only four out of the 61 analyzed candidates, 3 of which belonged to the Euryarchaeota phylum and only one branched exclusively with sequences from Methanococcales. The bulk of the genes branched with bacterial sequences, from which 12 had no clear association with any phylum. Bacteroidetes was the most prevalent donor group with sixteen donated genes; moreover, ten of them were probably transferred from the order Bacteroidales. In 6 trees the* Entamoeba* genes branched inside a larger cluster with high posterior probability values. Alternatively, in 9 other cases amoebic genes were separated from their basal group by a large evolutionary distance, but being branched with their sister group always showed high supporting values. The second most abundant donor group was the phylum Firmicutes, even though only in 4—out of the eleven candidates—a donor order could be designated. One gene branched strongly with sequences from Bacillales and the other 3 branched with Clostridiales. In most cases, the posterior probability of every node between the* Entamoeba* genes and their sister group was close to 1.0, with the exception of the type A flavoprotein (EHI_09671), which grouped with several bacterial clusters through a polytomy. The phylum Proteobacteria was designated as the donor group for a total of seven genes; this was the phylum which presented the highest diversity of orders: Campylobacterales, Pseudomonadales, Burkholderiales, and Enterobacteriales. Despite the fact that just one gene belonged to each donor order, each one of them grouped with high posterior probability, with the exception of Fe-S cluster assembly protein (EHI_049620) whose posterior probability was 0.7 in the node between the amoebic cluster and sequences from Campylobacterales. Although one candidate branched poorly (posterior probability: 0.64) with sequences from Fusobacteria, it was not possible to determine a donor order. In up to 5 trees, the sequence of* E. invadens* did not branch with its* Entamoeba* ortholog but with prokaryotic sequences or as basal group instead.

### 3.3. Divergence Time Estimates

*Entamoeba* divergence time was calculated with the following set of five housekeeping genes: DNA-directed RNA polymerase I subunit RPA2, elongation factor 2, actin, tubulin gamma chain, and clathrin heavy chain. Orthologs from* E. histolytica*,* E. nuttallii*,* E. dispar*,* E. invadens*, and* D. discoideum* made up the dataset.

The median rate of molecular evolution among the five amoebic genes provided by Estbranches was 0.02364 substitutions per site per million years, which was then used as the mean and standard deviation of prior distribution for the rate of molecular evolution at the in-group root node for the Multidivtime program. Finally, the estimates for the split ages between these lineages were the following: split date between* E. nuttallii* and* E. histolytica*, 5.93 ± 0.28 Mya, between* E. histolytica* and* E. dispar*, 9.97 ± 1.37 Mya, and between* E. histolytica* and* E. invadens*, 68.18 ± 16.04 Mya (Figure 3).

**Figure 2:**
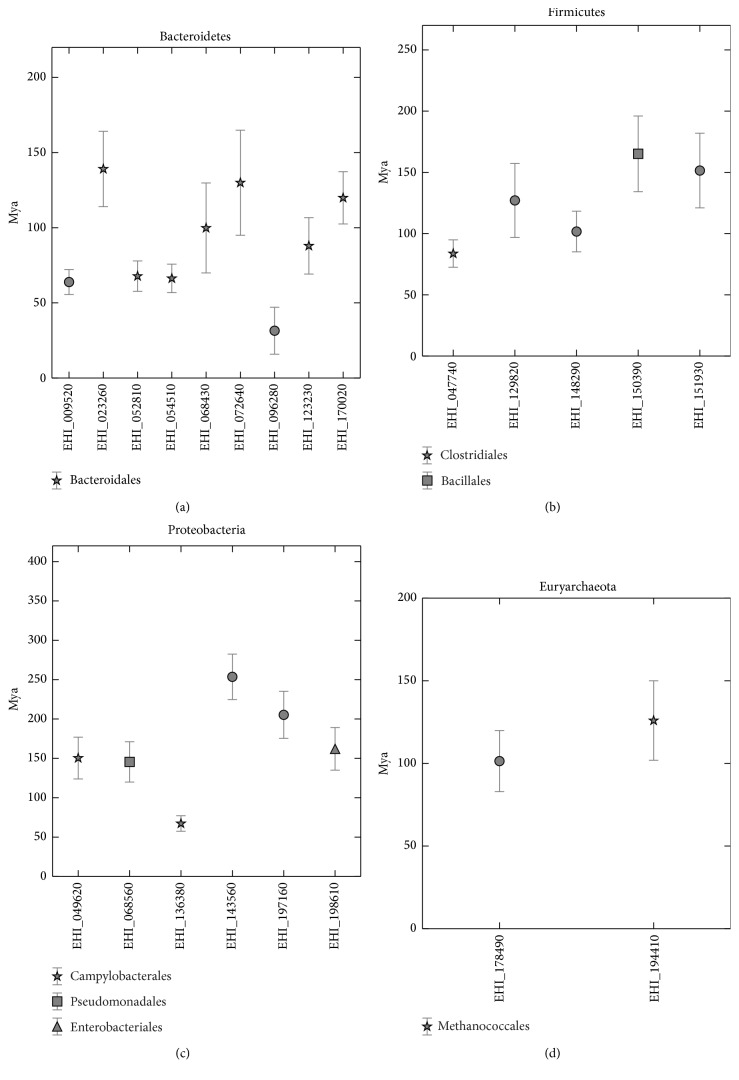
Times of horizontal gene transfer by phylogenetic groups. Measures of dispersion by standard deviation. Some simultaneous events specifically in group of Bacteroidales can be proposed.

Twenty-two* Entamoeba* candidates were selected after considering three criteria: well supported branching in the Bayesian phylogenies, assigned donor group at least at the phylum level, and the presence of an ortholog in* E. nuttallii*. The most recent transference was that of gene endo-1,4-beta-xylanase (EHI_096280) from Bacteroidetes dated 31.45 ± 15.69 Mya. The oldest transferences occurred 253.59 ± 28.91 Mya when the gene tartrate dehydrogenase (EHI_143560) was donated by Proteobacteria (Figure 2). The median of molecular evolution for this gene was 0.00089 substitutions per site per million years; interestingly, this rate is smaller than the final rate of substitutions for the five amoebic housekeeping genes, which was 0.0014 substitutions per site per million years. This slow rate explains why such an old HGT event is still detectable and also why a donor group could still be determined for this gene. This is interesting because it is possible that some other HGT events may have been masked because of higher nucleotide substitution rates and the homogenization of the xenolog gene to the recipient genome.

Several overlapping transference dates were found, some of them from the same donor group: alpha-1,2-mannosidase (EHI_009520), mannose-1-phosphate guanylyltransferase (EHI_052810), and fructokinase (EHI_054510) from Bacteroidetes ranging from 55.53 Mya to 77.8715 Mya, nicotinate-phosphoribosyltransferase (EHI_023260) and hypothetical protein (EHI_072640) from Bacteroidales ranging from 94.98 Mya to 164.16 Mya, and Fe-S cluster assembly protein NifU (EHI_049620) and metallo-beta-lactamase family protein (EHI_068560) from Proteobacteria ranging from 119.89 Mya to 176.9304 Mya. Gene synteny in the genome of* E. histolytica* and functional group information were still necessary to define simultaneous horizontal gene transfer events.

## 4. Discussion

In this study the substitution saturation of the well supported HGT gene candidates from the genome of* E. histolytica* was verified and assigned a putative donor group for each candidate through phylogenetic reconstruction. In addition, a first approach into the divergence time estimation of some species of* Entamoeba* through indirect node calibration was presented, using the fossil record of their feasible hosts. Finally the gene transfer events of some HGT candidates were dated, revealing gene losses, postdivergence transfers, and a simultaneous transfer of two genes. The BLAST search results were able to provide a glimpse of the analysis outcome, since the top hits that resulted in the highest *e*-values (*e*-13 and *e*-16 for EHI_085050 and EHI_156240, resp.) belonged to candidates discarded because of substitution saturation. Moreover, for the 3 candidates whose top hits were sequences from Archaea, the latter domain was the putative donor group after inspecting the Bayesian phylogenies. It is interesting that most of the* Entamoeba* HGT candidate genes have no or few paralogs in the genome of the different species of* Entamoeba* included in the analyses, while some others had at least 3 paralogs in each genome. The former diversification may be result of neutral evolution in the case of the gene that encodes the metallo-beta-lactamase superfamily protein (EHI_115720), considering that beta-lactam antibiotics induce bacterial cell wall degradation and therefore are innocuous to* Entamoeba* species [43]. On the other hand, two of the candidates with the largest gene families, hypothetical protein and aldehyde-alcohol dehydrogenase 2 (EHI_104900 and EHI_160940, resp.), were discarded after the substitution saturation tests. It is likely that these gene families are result of an ancient HGT and have lost phylogenetic information, due to mutational saturation; or they have been acquired through vertical descent and they are similar to bacterial sequences, as a result of the same mechanism.

The procedures here presented managed to find donor groups at the order level, including the following: Bacteroidales, Clostridiales, Spirochaetales, Campylobacterales, Burkholderiales, Bacillales, Flavobacteriales, Methanococcales, and Enterobacteriales. Most of the donor taxa can be found in the gut of vertebrates, it is well known that the three bacterial phyla are major part of the gut microbiota, but other less abundant groups have donated genetic material to* Entamoeba* species such as anaerobic Archaea. Although most members of the* Entamoeba* genus are parasitic or commensal organisms, lineages of free living* Entamoeba* like* E. ecuadoriensis* have been found [1], and some of these xenolog genes have been acquired from free living donor groups.

The Bacteroidetes phylum monopolizes lateral gene donations to the* Entamoeba* species included in this analysis, and the second most abundant group is the phylum Firmicutes and then Proteobacteria (Figure 1). These results are in contrast with those reported previously [44], in which they found 16 candidates closely related to Proteobacteria; nevertheless we found only 8. Likewise, we found no traces of HGT from Actinobacteria. These differences may result of the increased sampling due to the growth of biological databases and the substitution saturation tests. Consistent results are the frequency of LGT from Bacteroidetes, Spirochaetes, and Fusobacteria. In fact, the phylum Bacteroidetes has been found as potential donor to other horizontally transferred genes in different organisms, such as Ciliates [45] and Dinoflagellates [46], thus confirming the promiscuity of this taxon.

Several studies have highlighted the ecological relationship between* E. histolytica* and bacteria, specifically during pathogenesis [47, 48]. This study supports the importance of these associations as they can provide evolutionary innovations to the genus, and although no virulence factors have been transferred, antibiotic resistance genes are among the 61 candidates. In fact, the gene 5-nitroimidazole antibiotic resistance protein (EHI_068430) has been transferred from Bacteroidales. As most HGT events were millions of years ago, it is unlikely that these genes have functioned as acquired adaptations against antibiotics. These genes might have had other functions such as secondary metabolite degradation or no function at all, before the antibiotics were a selective pressure in the human gut.

Although the alignment for protein serine acetyltransferase (EHI_202040) resulted in little saturation while excluding the third position of each codon and its phylogeny showed a symmetric topology, it is very unlikely that an ancestor of the* Entamoeba* genus had obtained this from halophilic archaea and probably other bacteria group can be the donor.

Since estimating the age of the gene transfer events was one of the main objectives of this study, it was necessary to approximate the divergence times of the species of* Entamoeba*. To accomplish this aim, the identification of* E. nuttallii* as a separate lineage provided crucial information. The fact that* E. nuttallii* has only been isolated from rhesus macaques and* E. histolytica* has been found in feces from wild baboons [38, 39] led to the assumption that the* E. nuttallii* and* E. histolytica* lineages were separated simultaneously with their hosts at some time between 4 and 8 Mya according to the fossil record, although it has been assumed that this interval is narrower [49]. The results from the amoebic species split date calculations might be underestimated by the fact that only one node was calibrated, as there is no direct fossil record of species of* Entamoeba*. Although other authors have made studies regarding the age of the Amoebozoa phylum as a whole using animal and plant fossils, it was not possible to use their results because of differences in time scale and classification [13]. Some divergence time estimates of HGT candidates have overlapping standard deviations; this is particularly interesting when the donor groups coincide, because these genes could have been transferred at the same time. To determine if these genes were transferred simultaneously, some characteristics were taken into account: gene transfer age, donor group, metabolic context, and location in the genome. It has been suggested that functionally related genes might be located closely especially in prokaryotic genomes [50], and it should be expected that a simultaneous horizontal gene transfer would result in one or more xenologs positioned near one to another and functionally related. Two genes shared most of these attributes: the genes for the mannose-1-phosphate guanylyltransferase (EHI_052810) and the fructokinase (EHI_054510) were donated by Bacteroidales, probably between 60 and 70 million years in the past. Both genes are involved in fructose, mannose, amino sugar, and nucleotide sugar metabolism.

**Figure 3:**
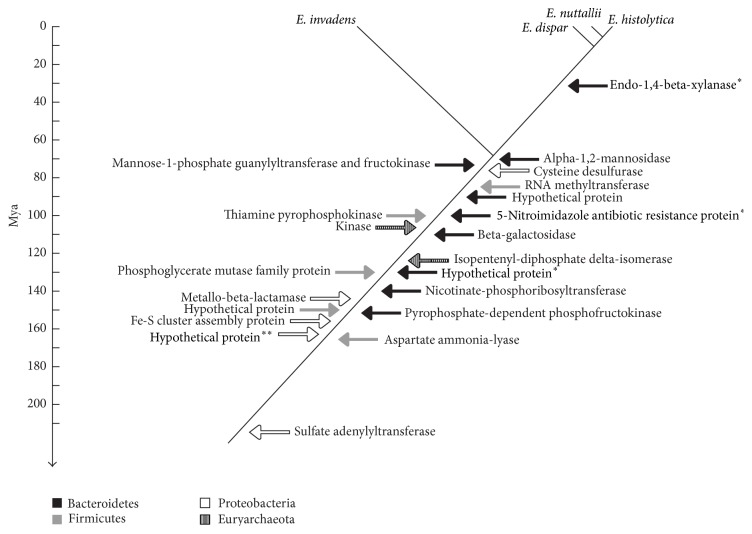
Divergence times of* Entamoeba* species, as well as horizontal transfers events. The units are Map (million years ago). ^*∗*^Genes without counterparts in the genome of* E. invadens*. ^*∗∗*^No homologous genes in the genome of* E. dispar*.

The dating of some transfers explained why certain genes are absent in some of the genomes of the amoebic species, included in this analysis (Figure 3). The transfer of the endo-1,4-beta-xylanase (EHI_096280) occurring 31.45 ± 15.69 Mya is in fact more recent than the divergence between the lineage of* E. invadens* and the ancestor of the other species of* Entamoeba* (68.18 ± 16.04 Mya); thus this gene was never present in the genome of the immediate ancestor of* E. invadens*. Conversely, the coding sequence for the 5-nitroimidazole-resistance protein (EHI_068430), which was transferred earlier (99.82 ± 29.94 Mya), was lost afterwards by* E. invadens*. The same conclusion could be applied to the gene coding for the hypothetic protein (EHI_198610), which was obtained 162.08 ± 27.07 Mya from Proteobacteria, which is now absent in the genome of* E. dispar*.

*E. histolytica* remains as one of the protists with the highest number of laterally transferred genes from bacterial origin in their genomes along with* Trichomonas vaginalis* and* Giardia lamblia* [9] or along with* Leishmania mayor* and* Trypanosoma brucei* [19]. This large uptake of bacterial genes, which in general took place relatively early in the evolutionary history of the* Entamoeba* genus, may have functioned as a trigger for adaptive evolution. The latter assertion may be palpable in the case of the genes coding for the acetyl-CoA synthetase and the adh1; but other genes gained through HGT, whose functions are unknown or obscured by biased annotations, may have been also important in the evolution of these organisms. The ancestor of the genus* Entamoeba*, which in our point of view, might as well be the ancestor of* Endolimax*, equipped with this newly acquired genes, might have tried exploring new ecosystems and forms of life and eventually settled in the gut of vertebrates.

## Supplementary Material

The supplementary information consists of image files showing the consensus trees built for this research. Files are named with the AmoebaDB accession number of the horizontal gene transfer can-didate being tested. Files with the “_tre_ed.png” suffix are the trees built to evaluate the phylogenetic relevance of shorter alignments, these trees were built with the MrBayes 3.2 software. The posterior probability is shown for each node and the bar shows the expected number of substitutions. The re-maining files show the consensus trees generated for the designations of donor groups. These are the consensus topologies returned by the program MrBayes 3.2, showing in each node, its posterior prob-ability. Whenever a tree built with Phyml showed the same node, the bootstrap value was added man-ually. The bar shows the number of expected substitutions. Image files were generated and edited using the program Dendroscope.
