# Supplementary figures and images for: Horizontal Gene Transfers from Bacteria to Entamoeba Complex: A Strategy for Dating Events along Species Divergence
Source: J Parasitol Res. 2016 Apr 27;2016:3241027. doi: 10.1155/2016/3241027 (PMC4863120; doi:10.1155/2016/3241027)

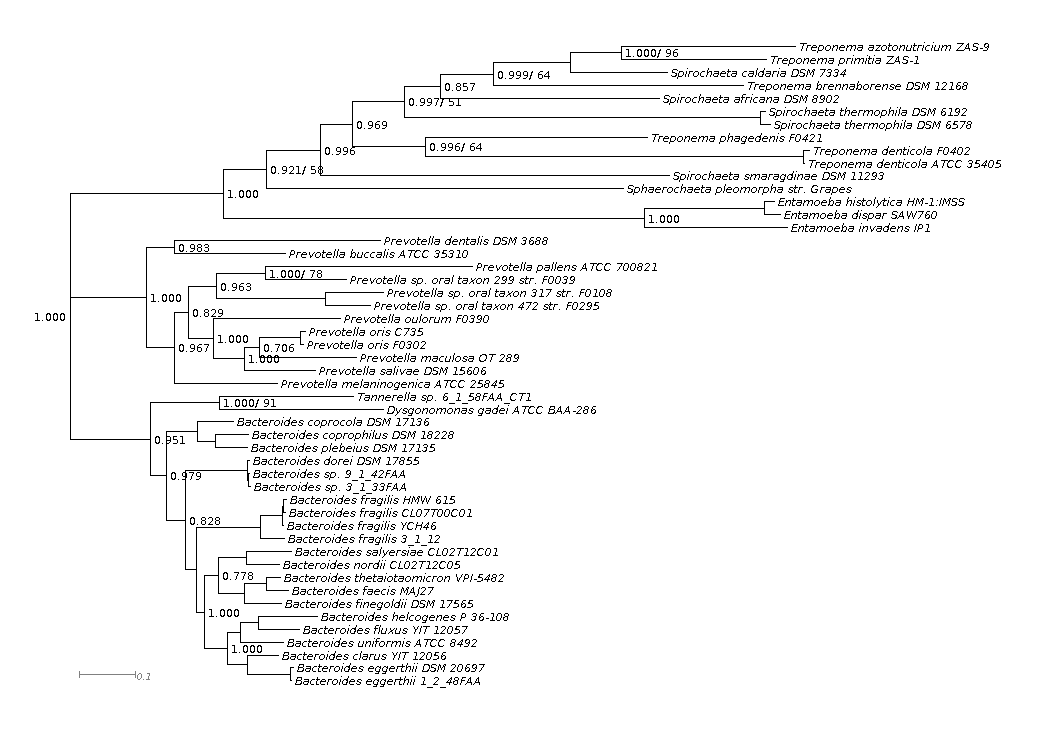

Supplement: Supplementary file 1 — The supplementary information consists of image files showing the consensus trees built for this research. Files are named with the AmoebaDB accession number of the horizontal gene transfer can-didate being tested. Files with the “_tre_ed.png” suffix are the trees built to evaluate the phylogenetic relevance of shorter alignments, these trees were built with the MrBayes 3.2 software. The posterior probability is shown for each node and the bar shows the expected number of substitutions. The re-maining files show the consensus trees generated for the designations of donor groups. These are the consensus topologies returned by the program MrBayes 3.2, showing in each node, its posterior prob-ability. Whenever a tree built with Phyml showed the same node, the bootstrap value was added man-ually. The bar shows the number of expected substitutions. Image files were generated and edited using the program Dendroscope. [file 3241027.f1.zip › EHI_000730_.png]

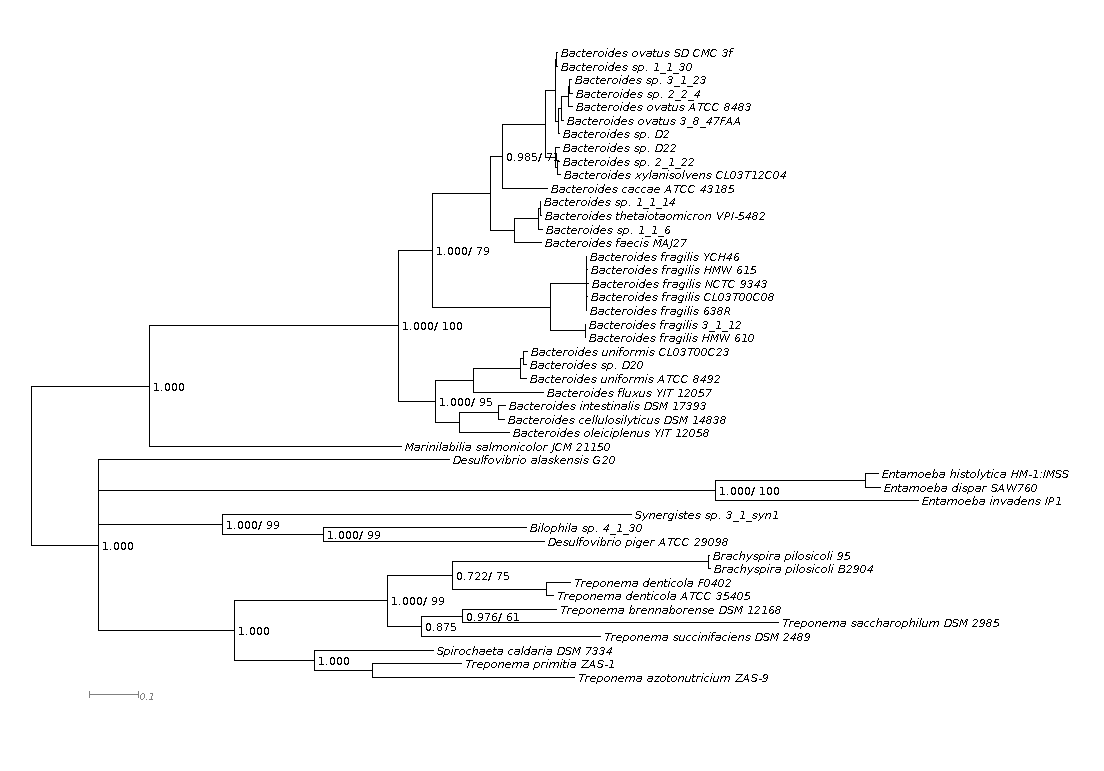

Supplement: Supplementary file 1 — The supplementary information consists of image files showing the consensus trees built for this research. Files are named with the AmoebaDB accession number of the horizontal gene transfer can-didate being tested. Files with the “_tre_ed.png” suffix are the trees built to evaluate the phylogenetic relevance of shorter alignments, these trees were built with the MrBayes 3.2 software. The posterior probability is shown for each node and the bar shows the expected number of substitutions. The re-maining files show the consensus trees generated for the designations of donor groups. These are the consensus topologies returned by the program MrBayes 3.2, showing in each node, its posterior prob-ability. Whenever a tree built with Phyml showed the same node, the bootstrap value was added man-ually. The bar shows the number of expected substitutions. Image files were generated and edited using the program Dendroscope. [file 3241027.f1.zip › EHI_005060_.png]

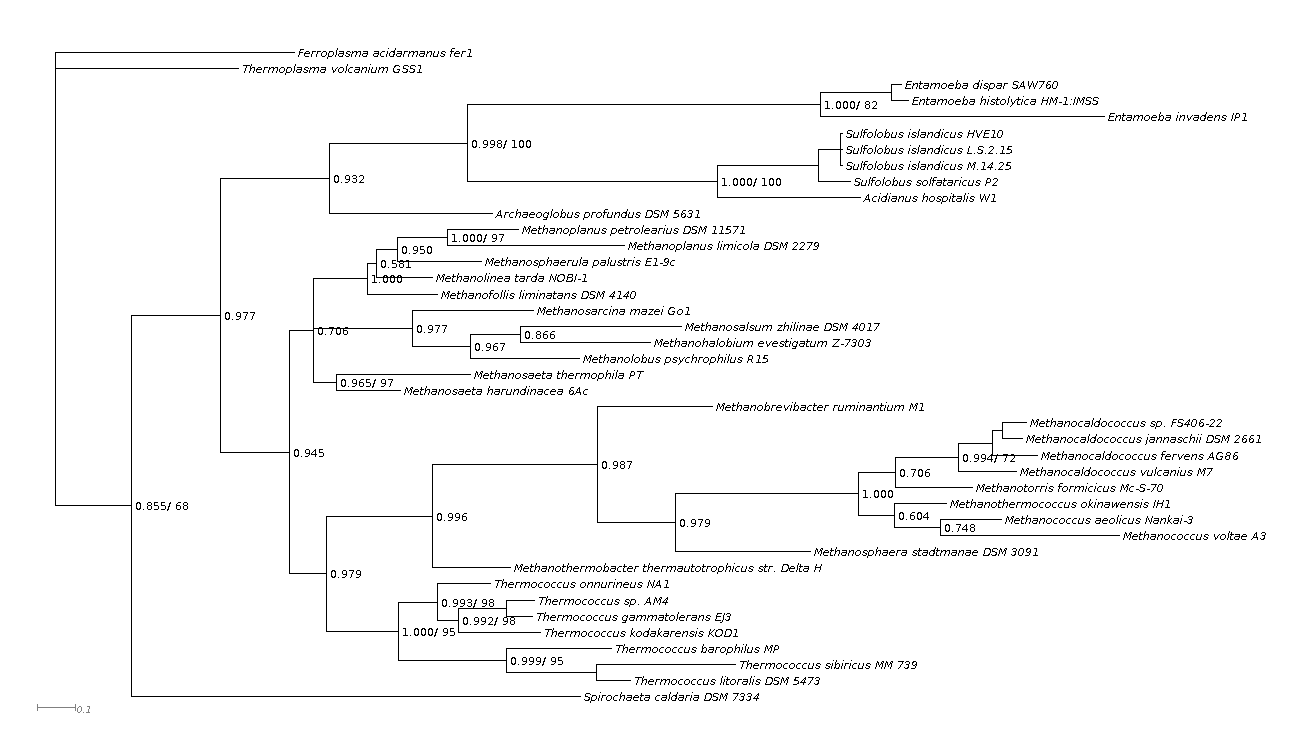

Supplement: Supplementary file 1 — The supplementary information consists of image files showing the consensus trees built for this research. Files are named with the AmoebaDB accession number of the horizontal gene transfer can-didate being tested. Files with the “_tre_ed.png” suffix are the trees built to evaluate the phylogenetic relevance of shorter alignments, these trees were built with the MrBayes 3.2 software. The posterior probability is shown for each node and the bar shows the expected number of substitutions. The re-maining files show the consensus trees generated for the designations of donor groups. These are the consensus topologies returned by the program MrBayes 3.2, showing in each node, its posterior prob-ability. Whenever a tree built with Phyml showed the same node, the bootstrap value was added man-ually. The bar shows the number of expected substitutions. Image files were generated and edited using the program Dendroscope. [file 3241027.f1.zip › EHI_006720_.png]

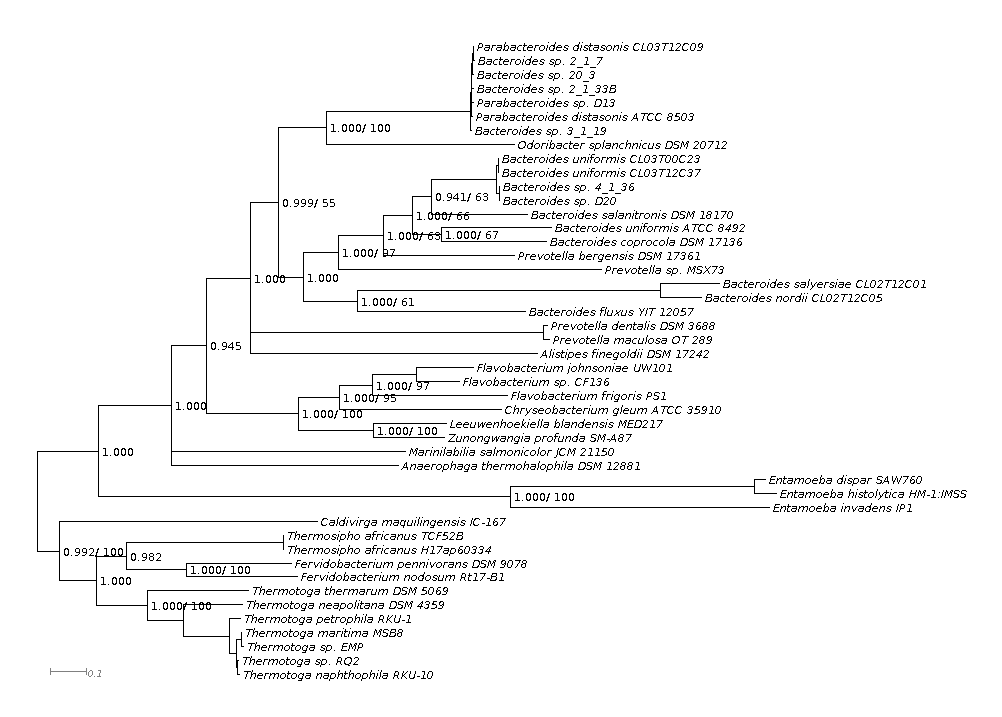

Supplement: Supplementary file 1 — The supplementary information consists of image files showing the consensus trees built for this research. Files are named with the AmoebaDB accession number of the horizontal gene transfer can-didate being tested. Files with the “_tre_ed.png” suffix are the trees built to evaluate the phylogenetic relevance of shorter alignments, these trees were built with the MrBayes 3.2 software. The posterior probability is shown for each node and the bar shows the expected number of substitutions. The re-maining files show the consensus trees generated for the designations of donor groups. These are the consensus topologies returned by the program MrBayes 3.2, showing in each node, its posterior prob-ability. Whenever a tree built with Phyml showed the same node, the bootstrap value was added man-ually. The bar shows the number of expected substitutions. Image files were generated and edited using the program Dendroscope. [file 3241027.f1.zip › EHI_007880_.png]

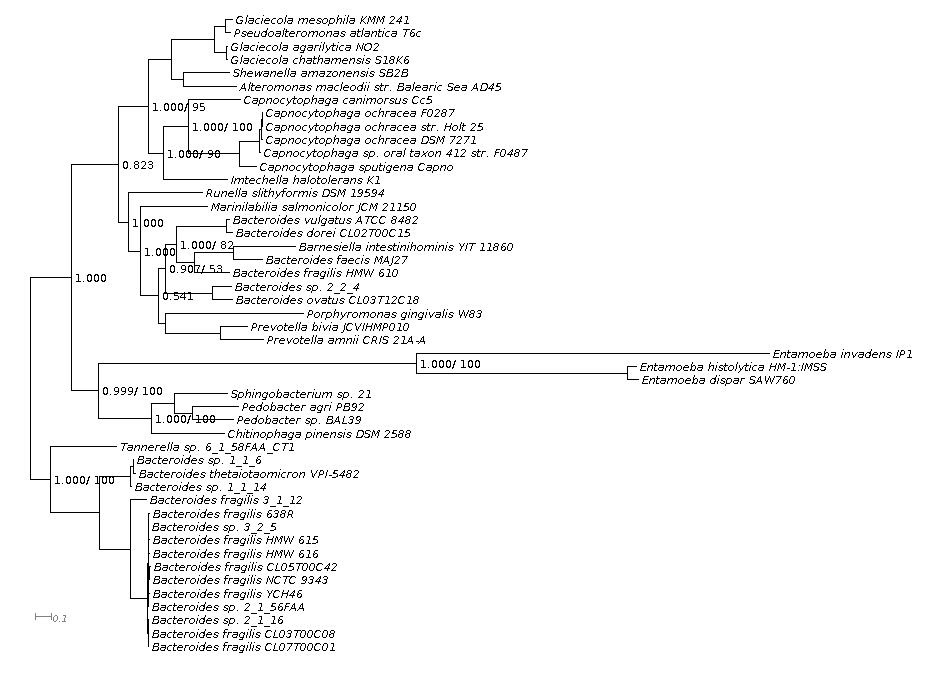

Supplement: Supplementary file 1 — The supplementary information consists of image files showing the consensus trees built for this research. Files are named with the AmoebaDB accession number of the horizontal gene transfer can-didate being tested. Files with the “_tre_ed.png” suffix are the trees built to evaluate the phylogenetic relevance of shorter alignments, these trees were built with the MrBayes 3.2 software. The posterior probability is shown for each node and the bar shows the expected number of substitutions. The re-maining files show the consensus trees generated for the designations of donor groups. These are the consensus topologies returned by the program MrBayes 3.2, showing in each node, its posterior prob-ability. Whenever a tree built with Phyml showed the same node, the bootstrap value was added man-ually. The bar shows the number of expected substitutions. Image files were generated and edited using the program Dendroscope. [file 3241027.f1.zip › EHI_009520_.png]

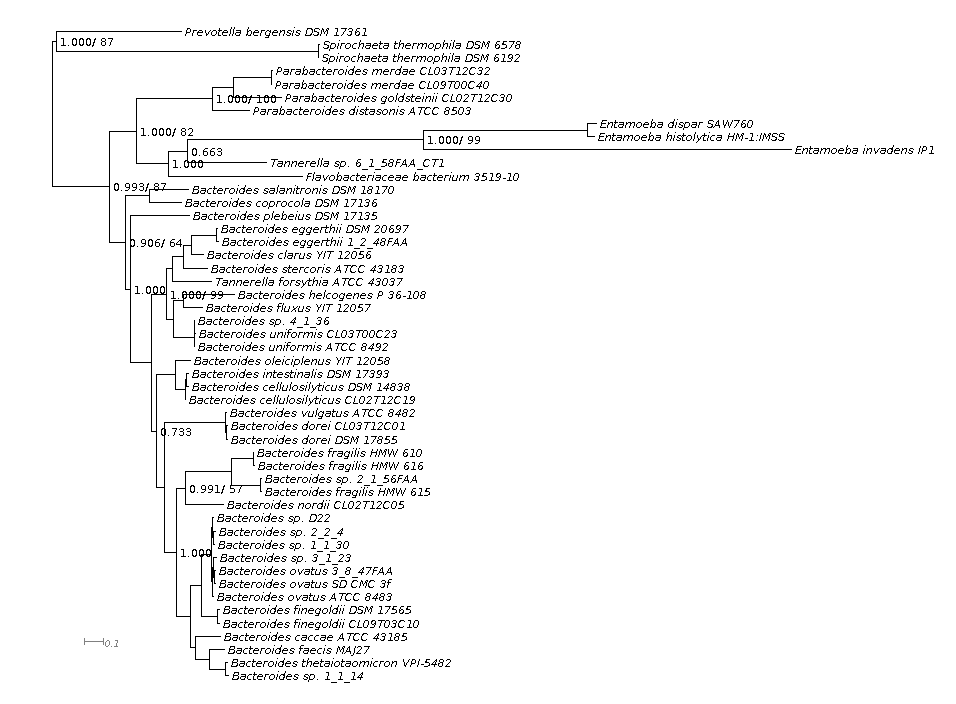

Supplement: Supplementary file 1 — The supplementary information consists of image files showing the consensus trees built for this research. Files are named with the AmoebaDB accession number of the horizontal gene transfer can-didate being tested. Files with the “_tre_ed.png” suffix are the trees built to evaluate the phylogenetic relevance of shorter alignments, these trees were built with the MrBayes 3.2 software. The posterior probability is shown for each node and the bar shows the expected number of substitutions. The re-maining files show the consensus trees generated for the designations of donor groups. These are the consensus topologies returned by the program MrBayes 3.2, showing in each node, its posterior prob-ability. Whenever a tree built with Phyml showed the same node, the bootstrap value was added man-ually. The bar shows the number of expected substitutions. Image files were generated and edited using the program Dendroscope. [file 3241027.f1.zip › EHI_012230_.png]

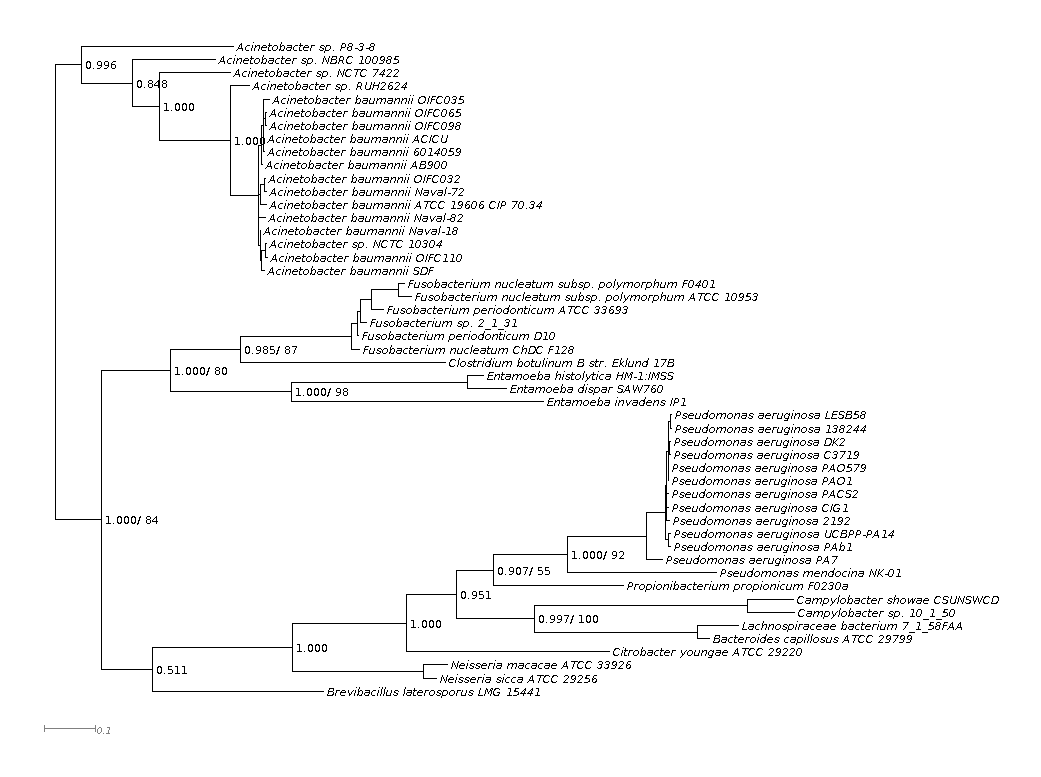

Supplement: Supplementary file 1 — The supplementary information consists of image files showing the consensus trees built for this research. Files are named with the AmoebaDB accession number of the horizontal gene transfer can-didate being tested. Files with the “_tre_ed.png” suffix are the trees built to evaluate the phylogenetic relevance of shorter alignments, these trees were built with the MrBayes 3.2 software. The posterior probability is shown for each node and the bar shows the expected number of substitutions. The re-maining files show the consensus trees generated for the designations of donor groups. These are the consensus topologies returned by the program MrBayes 3.2, showing in each node, its posterior prob-ability. Whenever a tree built with Phyml showed the same node, the bootstrap value was added man-ually. The bar shows the number of expected substitutions. Image files were generated and edited using the program Dendroscope. [file 3241027.f1.zip › EHI_017590_.png]

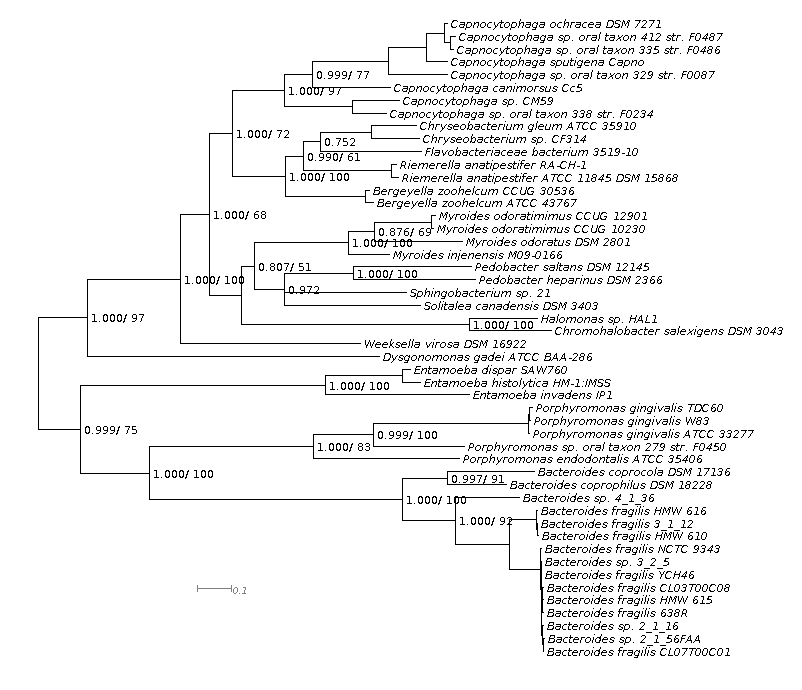

Supplement: Supplementary file 1 — The supplementary information consists of image files showing the consensus trees built for this research. Files are named with the AmoebaDB accession number of the horizontal gene transfer can-didate being tested. Files with the “_tre_ed.png” suffix are the trees built to evaluate the phylogenetic relevance of shorter alignments, these trees were built with the MrBayes 3.2 software. The posterior probability is shown for each node and the bar shows the expected number of substitutions. The re-maining files show the consensus trees generated for the designations of donor groups. These are the consensus topologies returned by the program MrBayes 3.2, showing in each node, its posterior prob-ability. Whenever a tree built with Phyml showed the same node, the bootstrap value was added man-ually. The bar shows the number of expected substitutions. Image files were generated and edited using the program Dendroscope. [file 3241027.f1.zip › EHI_023260_.png]

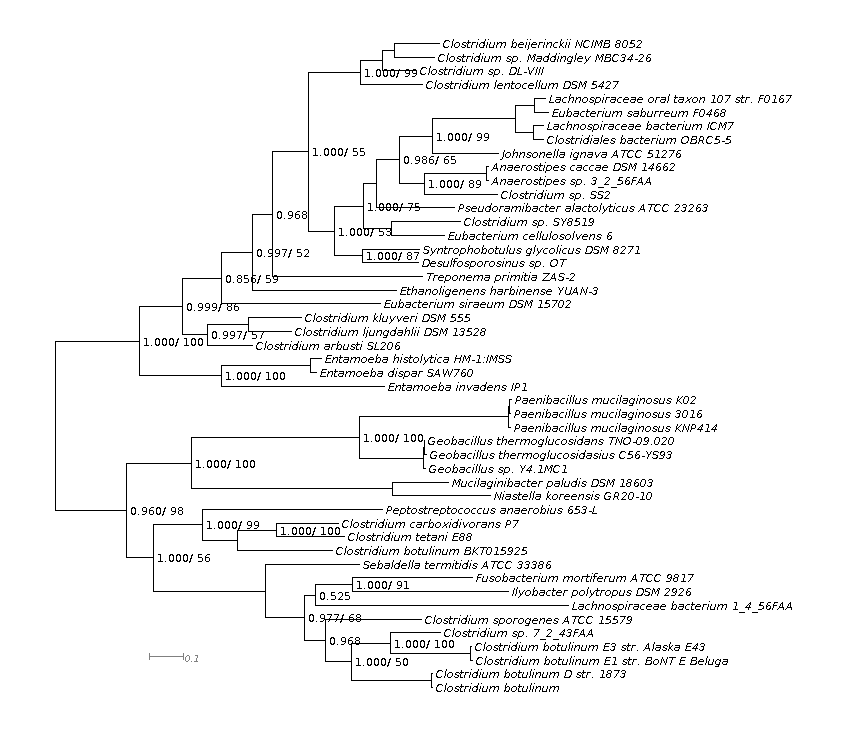

Supplement: Supplementary file 1 — The supplementary information consists of image files showing the consensus trees built for this research. Files are named with the AmoebaDB accession number of the horizontal gene transfer can-didate being tested. Files with the “_tre_ed.png” suffix are the trees built to evaluate the phylogenetic relevance of shorter alignments, these trees were built with the MrBayes 3.2 software. The posterior probability is shown for each node and the bar shows the expected number of substitutions. The re-maining files show the consensus trees generated for the designations of donor groups. These are the consensus topologies returned by the program MrBayes 3.2, showing in each node, its posterior prob-ability. Whenever a tree built with Phyml showed the same node, the bootstrap value was added man-ually. The bar shows the number of expected substitutions. Image files were generated and edited using the program Dendroscope. [file 3241027.f1.zip › EHI_024420_.png]

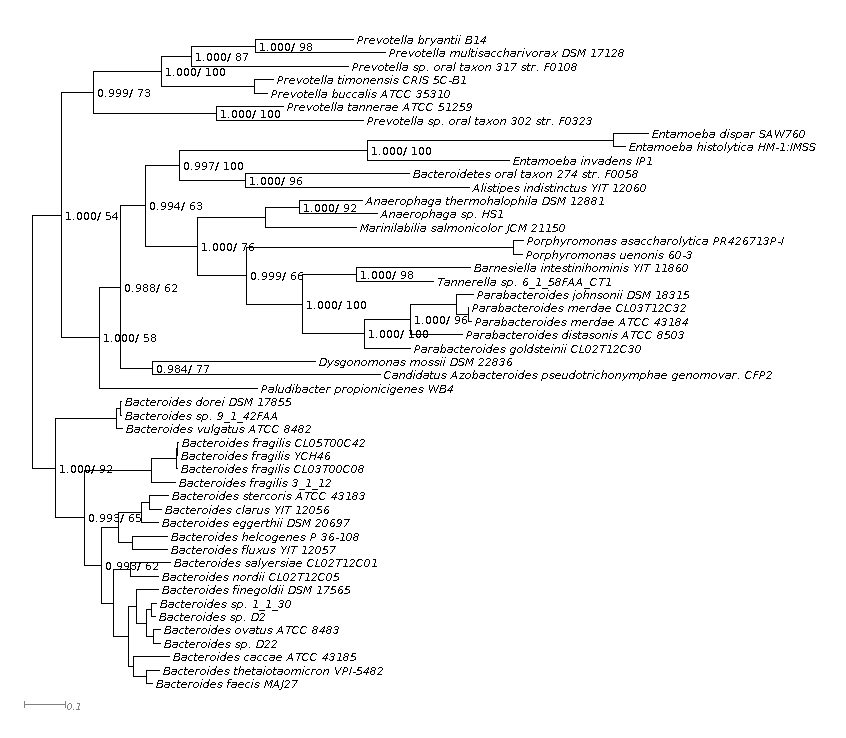

Supplement: Supplementary file 1 — The supplementary information consists of image files showing the consensus trees built for this research. Files are named with the AmoebaDB accession number of the horizontal gene transfer can-didate being tested. Files with the “_tre_ed.png” suffix are the trees built to evaluate the phylogenetic relevance of shorter alignments, these trees were built with the MrBayes 3.2 software. The posterior probability is shown for each node and the bar shows the expected number of substitutions. The re-maining files show the consensus trees generated for the designations of donor groups. These are the consensus topologies returned by the program MrBayes 3.2, showing in each node, its posterior prob-ability. Whenever a tree built with Phyml showed the same node, the bootstrap value was added man-ually. The bar shows the number of expected substitutions. Image files were generated and edited using the program Dendroscope. [file 3241027.f1.zip › EHI_026360_.png]

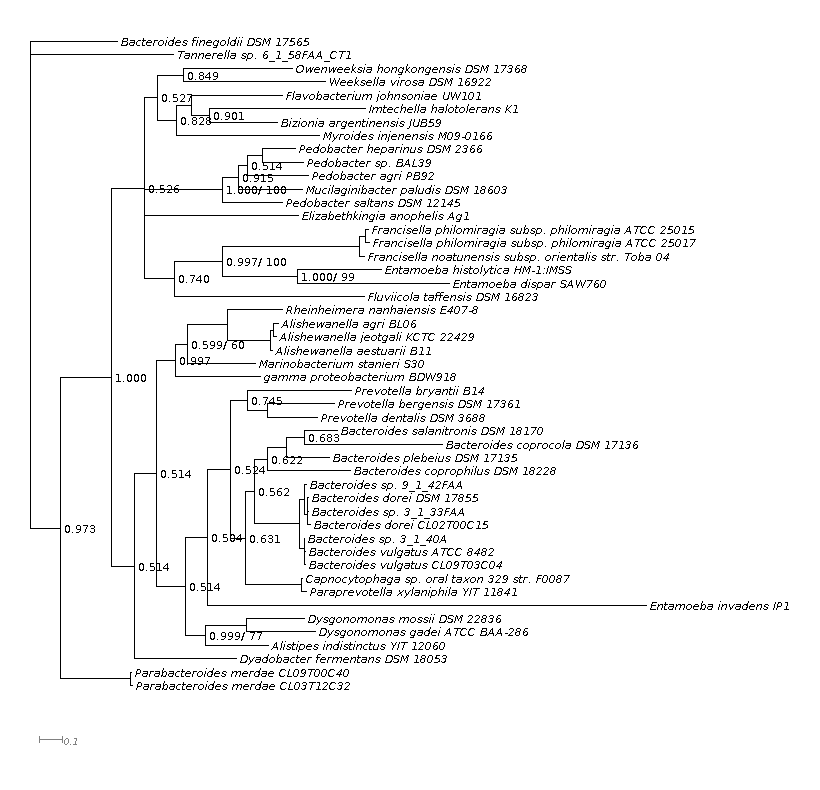

Supplement: Supplementary file 1 — The supplementary information consists of image files showing the consensus trees built for this research. Files are named with the AmoebaDB accession number of the horizontal gene transfer can-didate being tested. Files with the “_tre_ed.png” suffix are the trees built to evaluate the phylogenetic relevance of shorter alignments, these trees were built with the MrBayes 3.2 software. The posterior probability is shown for each node and the bar shows the expected number of substitutions. The re-maining files show the consensus trees generated for the designations of donor groups. These are the consensus topologies returned by the program MrBayes 3.2, showing in each node, its posterior prob-ability. Whenever a tree built with Phyml showed the same node, the bootstrap value was added man-ually. The bar shows the number of expected substitutions. Image files were generated and edited using the program Dendroscope. [file 3241027.f1.zip › EHI_029230_.png]

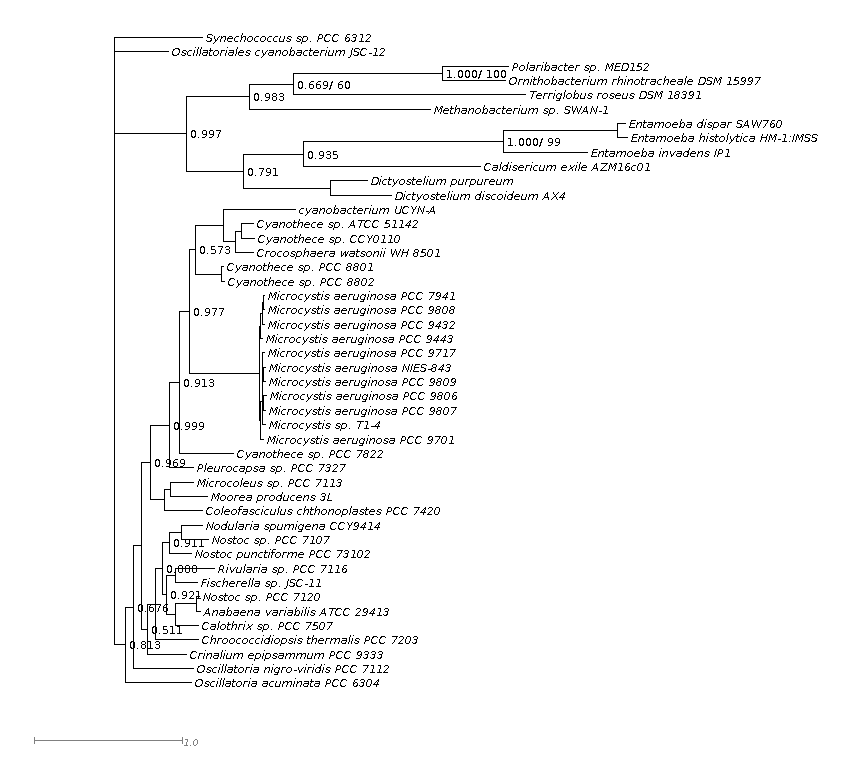

Supplement: Supplementary file 1 — The supplementary information consists of image files showing the consensus trees built for this research. Files are named with the AmoebaDB accession number of the horizontal gene transfer can-didate being tested. Files with the “_tre_ed.png” suffix are the trees built to evaluate the phylogenetic relevance of shorter alignments, these trees were built with the MrBayes 3.2 software. The posterior probability is shown for each node and the bar shows the expected number of substitutions. The re-maining files show the consensus trees generated for the designations of donor groups. These are the consensus topologies returned by the program MrBayes 3.2, showing in each node, its posterior prob-ability. Whenever a tree built with Phyml showed the same node, the bootstrap value was added man-ually. The bar shows the number of expected substitutions. Image files were generated and edited using the program Dendroscope. [file 3241027.f1.zip › EHI_035210_.png]

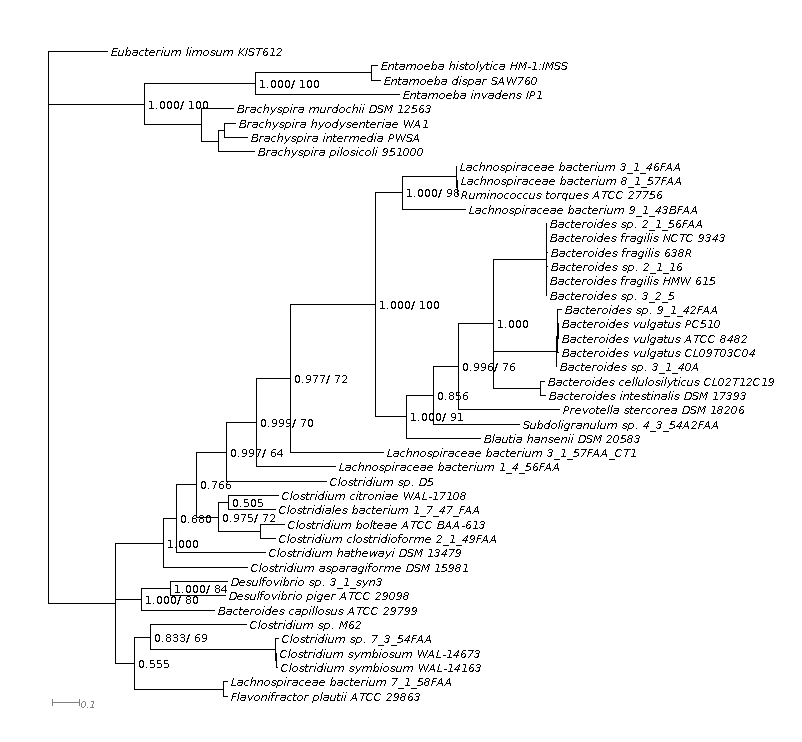

Supplement: Supplementary file 1 — The supplementary information consists of image files showing the consensus trees built for this research. Files are named with the AmoebaDB accession number of the horizontal gene transfer can-didate being tested. Files with the “_tre_ed.png” suffix are the trees built to evaluate the phylogenetic relevance of shorter alignments, these trees were built with the MrBayes 3.2 software. The posterior probability is shown for each node and the bar shows the expected number of substitutions. The re-maining files show the consensus trees generated for the designations of donor groups. These are the consensus topologies returned by the program MrBayes 3.2, showing in each node, its posterior prob-ability. Whenever a tree built with Phyml showed the same node, the bootstrap value was added man-ually. The bar shows the number of expected substitutions. Image files were generated and edited using the program Dendroscope. [file 3241027.f1.zip › EHI_042260_.png]

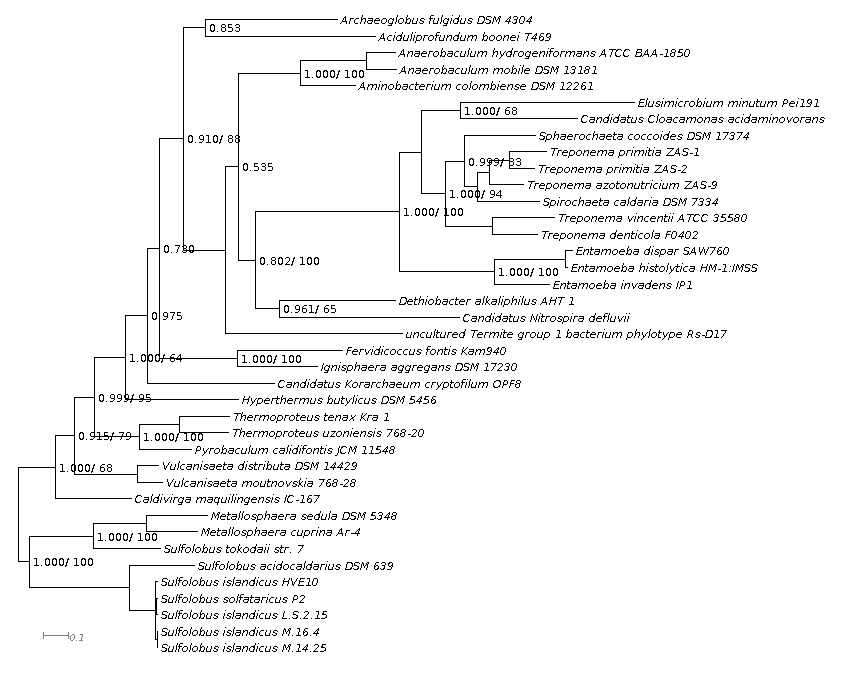

Supplement: Supplementary file 1 — The supplementary information consists of image files showing the consensus trees built for this research. Files are named with the AmoebaDB accession number of the horizontal gene transfer can-didate being tested. Files with the “_tre_ed.png” suffix are the trees built to evaluate the phylogenetic relevance of shorter alignments, these trees were built with the MrBayes 3.2 software. The posterior probability is shown for each node and the bar shows the expected number of substitutions. The re-maining files show the consensus trees generated for the designations of donor groups. These are the consensus topologies returned by the program MrBayes 3.2, showing in each node, its posterior prob-ability. Whenever a tree built with Phyml showed the same node, the bootstrap value was added man-ually. The bar shows the number of expected substitutions. Image files were generated and edited using the program Dendroscope. [file 3241027.f1.zip › EHI_044970_.png]

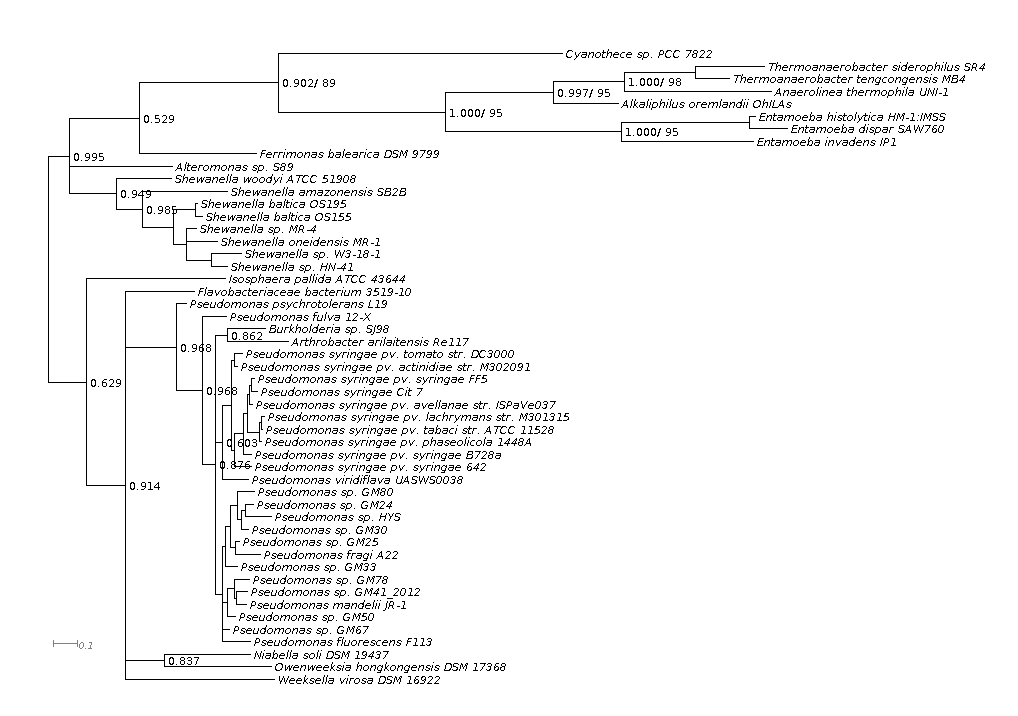

Supplement: Supplementary file 1 — The supplementary information consists of image files showing the consensus trees built for this research. Files are named with the AmoebaDB accession number of the horizontal gene transfer can-didate being tested. Files with the “_tre_ed.png” suffix are the trees built to evaluate the phylogenetic relevance of shorter alignments, these trees were built with the MrBayes 3.2 software. The posterior probability is shown for each node and the bar shows the expected number of substitutions. The re-maining files show the consensus trees generated for the designations of donor groups. These are the consensus topologies returned by the program MrBayes 3.2, showing in each node, its posterior prob-ability. Whenever a tree built with Phyml showed the same node, the bootstrap value was added man-ually. The bar shows the number of expected substitutions. Image files were generated and edited using the program Dendroscope. [file 3241027.f1.zip › EHI_046600_.png]

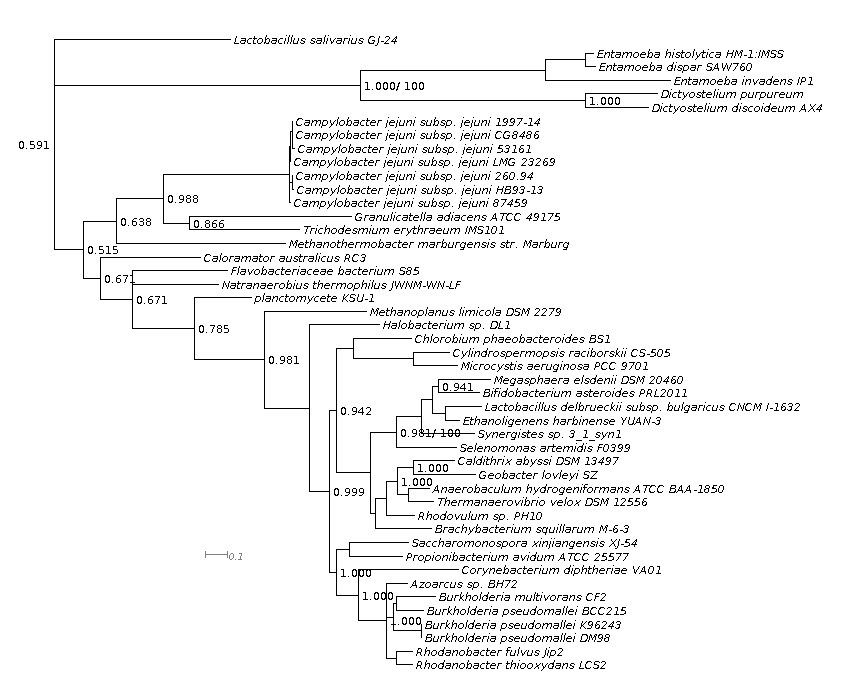

Supplement: Supplementary file 1 — The supplementary information consists of image files showing the consensus trees built for this research. Files are named with the AmoebaDB accession number of the horizontal gene transfer can-didate being tested. Files with the “_tre_ed.png” suffix are the trees built to evaluate the phylogenetic relevance of shorter alignments, these trees were built with the MrBayes 3.2 software. The posterior probability is shown for each node and the bar shows the expected number of substitutions. The re-maining files show the consensus trees generated for the designations of donor groups. These are the consensus topologies returned by the program MrBayes 3.2, showing in each node, its posterior prob-ability. Whenever a tree built with Phyml showed the same node, the bootstrap value was added man-ually. The bar shows the number of expected substitutions. Image files were generated and edited using the program Dendroscope. [file 3241027.f1.zip › EHI_046680_.png]

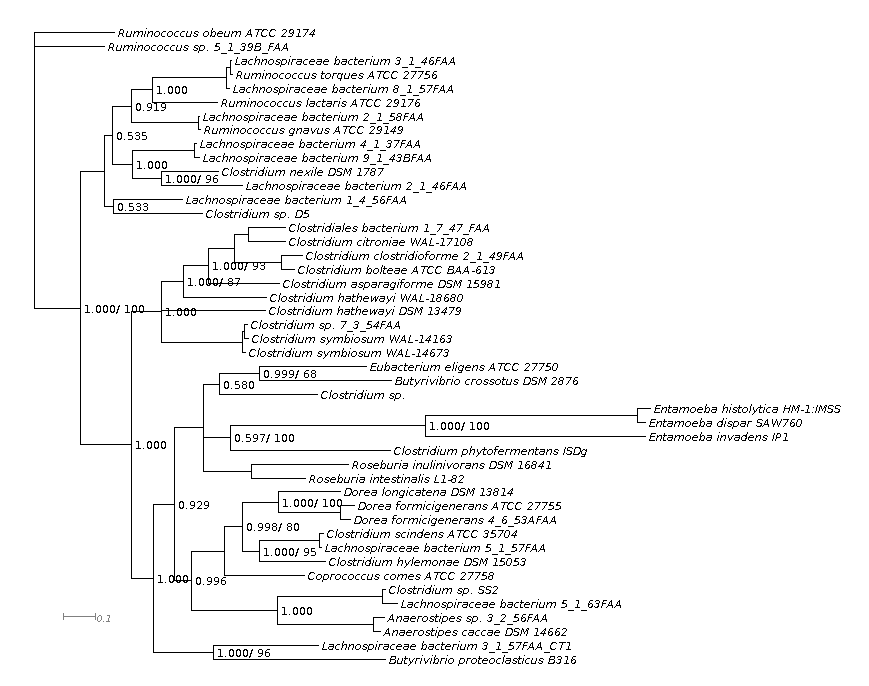

Supplement: Supplementary file 1 — The supplementary information consists of image files showing the consensus trees built for this research. Files are named with the AmoebaDB accession number of the horizontal gene transfer can-didate being tested. Files with the “_tre_ed.png” suffix are the trees built to evaluate the phylogenetic relevance of shorter alignments, these trees were built with the MrBayes 3.2 software. The posterior probability is shown for each node and the bar shows the expected number of substitutions. The re-maining files show the consensus trees generated for the designations of donor groups. These are the consensus topologies returned by the program MrBayes 3.2, showing in each node, its posterior prob-ability. Whenever a tree built with Phyml showed the same node, the bootstrap value was added man-ually. The bar shows the number of expected substitutions. Image files were generated and edited using the program Dendroscope. [file 3241027.f1.zip › EHI_047740_.png]

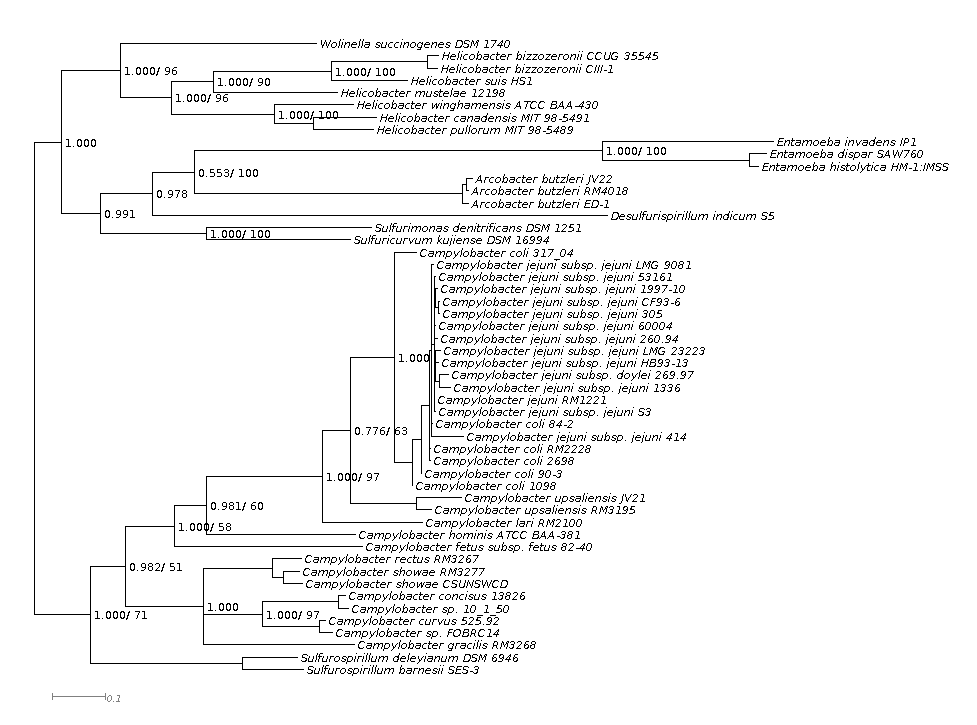

Supplement: Supplementary file 1 — The supplementary information consists of image files showing the consensus trees built for this research. Files are named with the AmoebaDB accession number of the horizontal gene transfer can-didate being tested. Files with the “_tre_ed.png” suffix are the trees built to evaluate the phylogenetic relevance of shorter alignments, these trees were built with the MrBayes 3.2 software. The posterior probability is shown for each node and the bar shows the expected number of substitutions. The re-maining files show the consensus trees generated for the designations of donor groups. These are the consensus topologies returned by the program MrBayes 3.2, showing in each node, its posterior prob-ability. Whenever a tree built with Phyml showed the same node, the bootstrap value was added man-ually. The bar shows the number of expected substitutions. Image files were generated and edited using the program Dendroscope. [file 3241027.f1.zip › EHI_049620_.png]

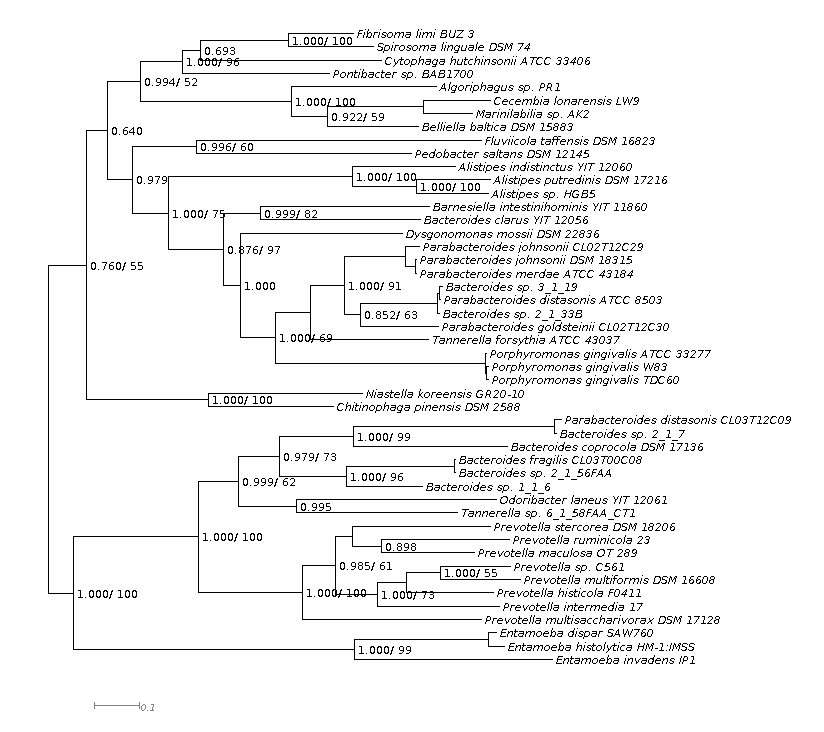

Supplement: Supplementary file 1 — The supplementary information consists of image files showing the consensus trees built for this research. Files are named with the AmoebaDB accession number of the horizontal gene transfer can-didate being tested. Files with the “_tre_ed.png” suffix are the trees built to evaluate the phylogenetic relevance of shorter alignments, these trees were built with the MrBayes 3.2 software. The posterior probability is shown for each node and the bar shows the expected number of substitutions. The re-maining files show the consensus trees generated for the designations of donor groups. These are the consensus topologies returned by the program MrBayes 3.2, showing in each node, its posterior prob-ability. Whenever a tree built with Phyml showed the same node, the bootstrap value was added man-ually. The bar shows the number of expected substitutions. Image files were generated and edited using the program Dendroscope. [file 3241027.f1.zip › EHI_052810_.png]

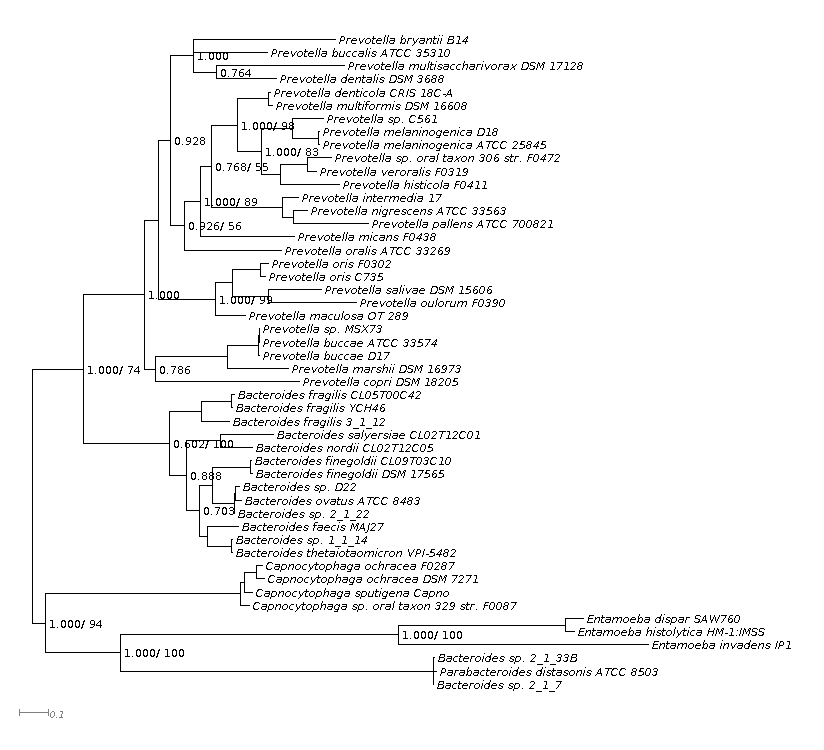

Supplement: Supplementary file 1 — The supplementary information consists of image files showing the consensus trees built for this research. Files are named with the AmoebaDB accession number of the horizontal gene transfer can-didate being tested. Files with the “_tre_ed.png” suffix are the trees built to evaluate the phylogenetic relevance of shorter alignments, these trees were built with the MrBayes 3.2 software. The posterior probability is shown for each node and the bar shows the expected number of substitutions. The re-maining files show the consensus trees generated for the designations of donor groups. These are the consensus topologies returned by the program MrBayes 3.2, showing in each node, its posterior prob-ability. Whenever a tree built with Phyml showed the same node, the bootstrap value was added man-ually. The bar shows the number of expected substitutions. Image files were generated and edited using the program Dendroscope. [file 3241027.f1.zip › EHI_054510_.png]

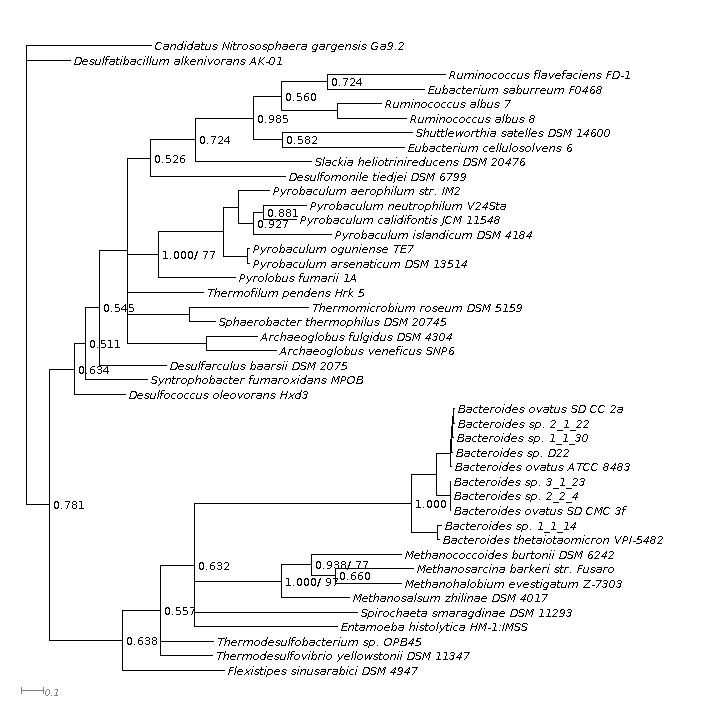

Supplement: Supplementary file 1 — The supplementary information consists of image files showing the consensus trees built for this research. Files are named with the AmoebaDB accession number of the horizontal gene transfer can-didate being tested. Files with the “_tre_ed.png” suffix are the trees built to evaluate the phylogenetic relevance of shorter alignments, these trees were built with the MrBayes 3.2 software. The posterior probability is shown for each node and the bar shows the expected number of substitutions. The re-maining files show the consensus trees generated for the designations of donor groups. These are the consensus topologies returned by the program MrBayes 3.2, showing in each node, its posterior prob-ability. Whenever a tree built with Phyml showed the same node, the bootstrap value was added man-ually. The bar shows the number of expected substitutions. Image files were generated and edited using the program Dendroscope. [file 3241027.f1.zip › EHI_054690_.png]

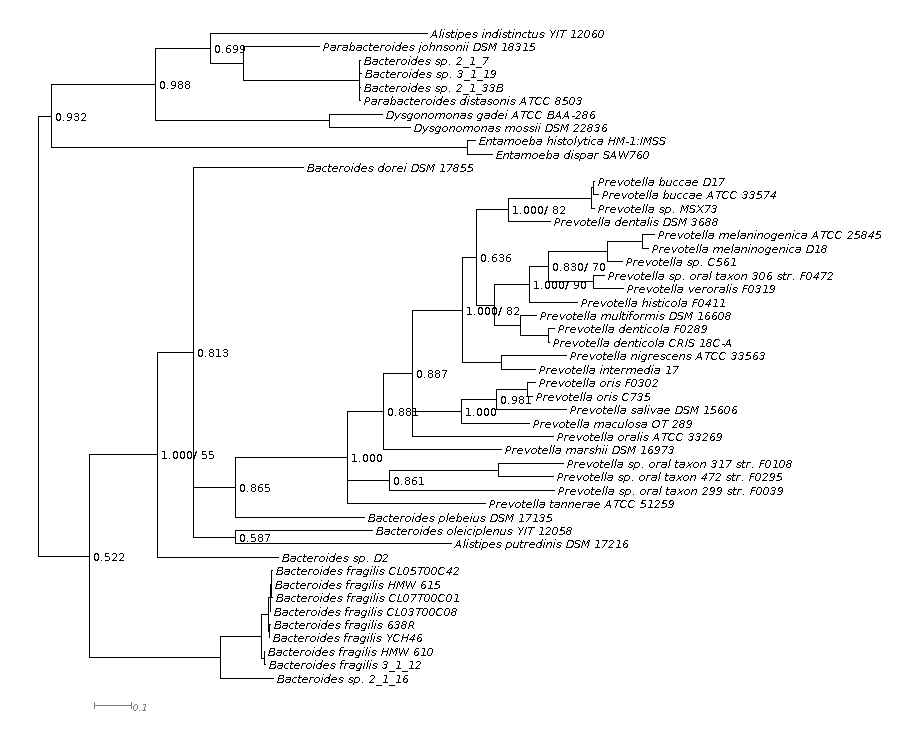

Supplement: Supplementary file 1 — The supplementary information consists of image files showing the consensus trees built for this research. Files are named with the AmoebaDB accession number of the horizontal gene transfer can-didate being tested. Files with the “_tre_ed.png” suffix are the trees built to evaluate the phylogenetic relevance of shorter alignments, these trees were built with the MrBayes 3.2 software. The posterior probability is shown for each node and the bar shows the expected number of substitutions. The re-maining files show the consensus trees generated for the designations of donor groups. These are the consensus topologies returned by the program MrBayes 3.2, showing in each node, its posterior prob-ability. Whenever a tree built with Phyml showed the same node, the bootstrap value was added man-ually. The bar shows the number of expected substitutions. Image files were generated and edited using the program Dendroscope. [file 3241027.f1.zip › EHI_068430_.png]

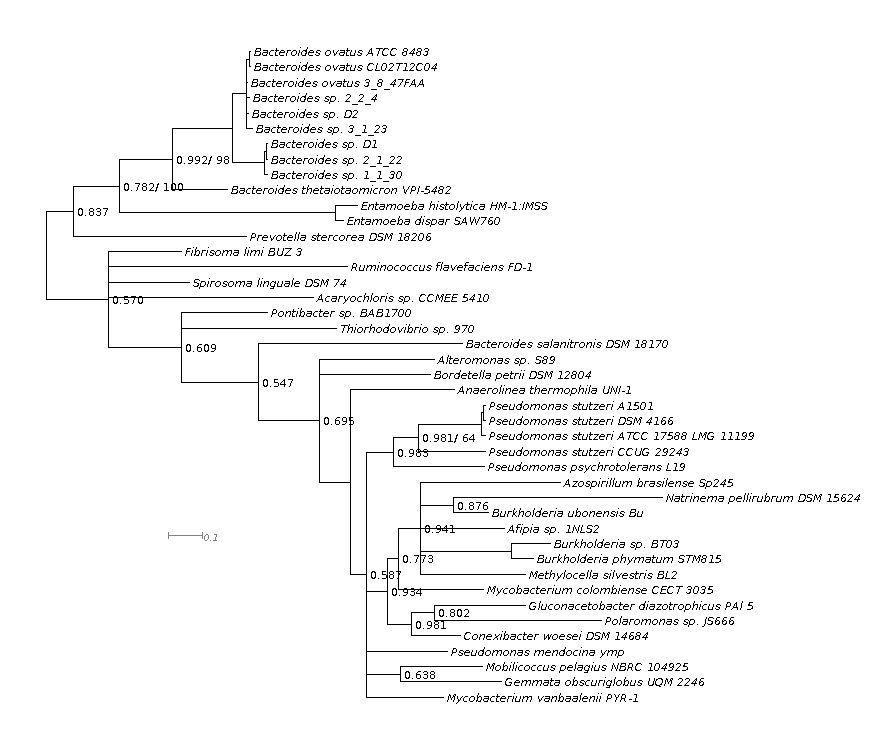

Supplement: Supplementary file 1 — The supplementary information consists of image files showing the consensus trees built for this research. Files are named with the AmoebaDB accession number of the horizontal gene transfer can-didate being tested. Files with the “_tre_ed.png” suffix are the trees built to evaluate the phylogenetic relevance of shorter alignments, these trees were built with the MrBayes 3.2 software. The posterior probability is shown for each node and the bar shows the expected number of substitutions. The re-maining files show the consensus trees generated for the designations of donor groups. These are the consensus topologies returned by the program MrBayes 3.2, showing in each node, its posterior prob-ability. Whenever a tree built with Phyml showed the same node, the bootstrap value was added man-ually. The bar shows the number of expected substitutions. Image files were generated and edited using the program Dendroscope. [file 3241027.f1.zip › EHI_072640_.png]

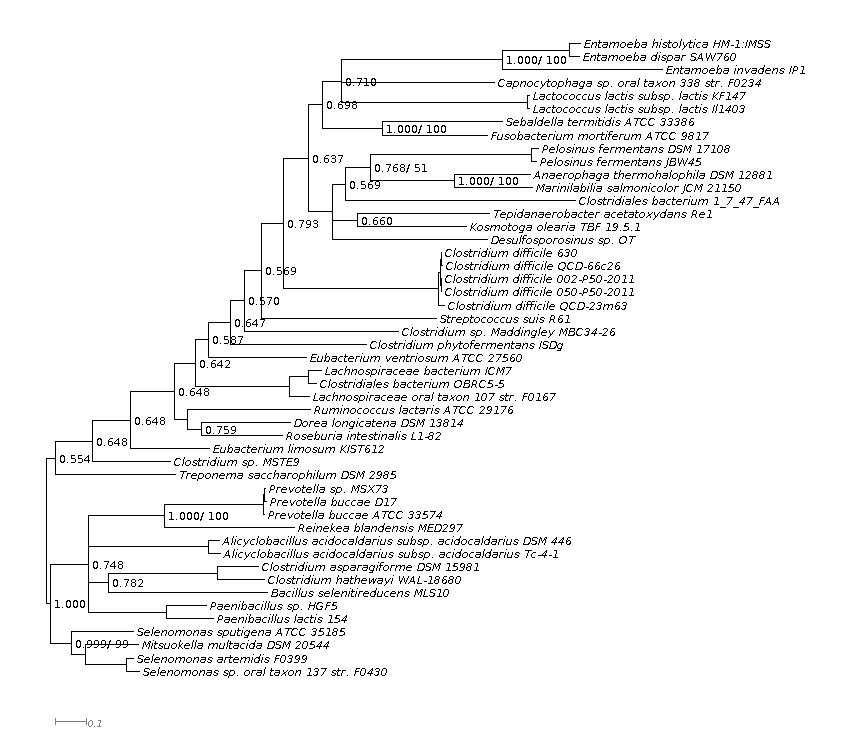

Supplement: Supplementary file 1 — The supplementary information consists of image files showing the consensus trees built for this research. Files are named with the AmoebaDB accession number of the horizontal gene transfer can-didate being tested. Files with the “_tre_ed.png” suffix are the trees built to evaluate the phylogenetic relevance of shorter alignments, these trees were built with the MrBayes 3.2 software. The posterior probability is shown for each node and the bar shows the expected number of substitutions. The re-maining files show the consensus trees generated for the designations of donor groups. These are the consensus topologies returned by the program MrBayes 3.2, showing in each node, its posterior prob-ability. Whenever a tree built with Phyml showed the same node, the bootstrap value was added man-ually. The bar shows the number of expected substitutions. Image files were generated and edited using the program Dendroscope. [file 3241027.f1.zip › EHI_084670_.png]

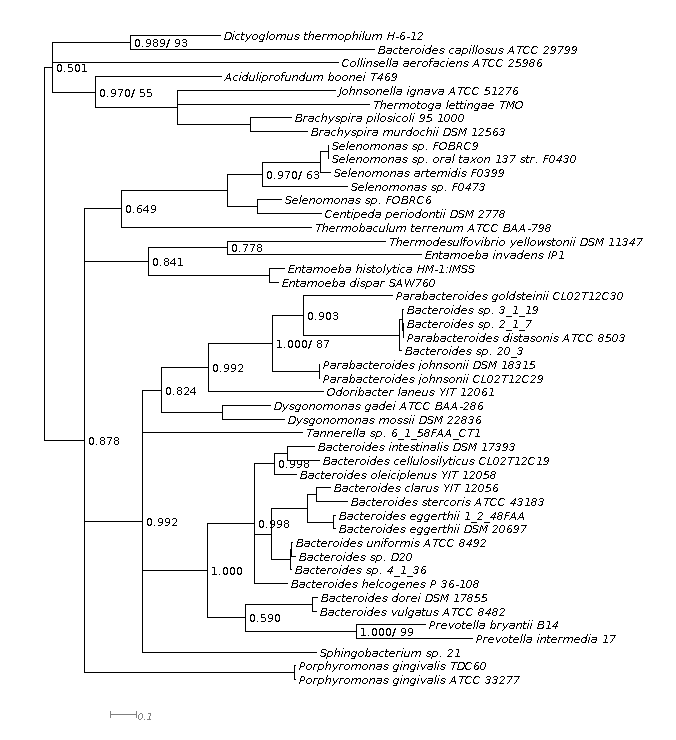

Supplement: Supplementary file 1 — The supplementary information consists of image files showing the consensus trees built for this research. Files are named with the AmoebaDB accession number of the horizontal gene transfer can-didate being tested. Files with the “_tre_ed.png” suffix are the trees built to evaluate the phylogenetic relevance of shorter alignments, these trees were built with the MrBayes 3.2 software. The posterior probability is shown for each node and the bar shows the expected number of substitutions. The re-maining files show the consensus trees generated for the designations of donor groups. These are the consensus topologies returned by the program MrBayes 3.2, showing in each node, its posterior prob-ability. Whenever a tree built with Phyml showed the same node, the bootstrap value was added man-ually. The bar shows the number of expected substitutions. Image files were generated and edited using the program Dendroscope. [file 3241027.f1.zip › EHI_090260_.png]

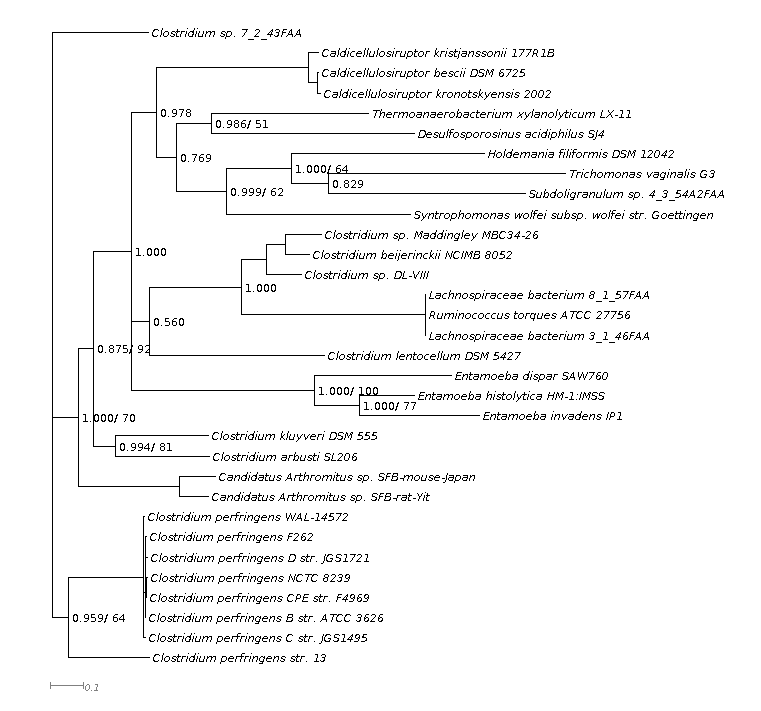

Supplement: Supplementary file 1 — The supplementary information consists of image files showing the consensus trees built for this research. Files are named with the AmoebaDB accession number of the horizontal gene transfer can-didate being tested. Files with the “_tre_ed.png” suffix are the trees built to evaluate the phylogenetic relevance of shorter alignments, these trees were built with the MrBayes 3.2 software. The posterior probability is shown for each node and the bar shows the expected number of substitutions. The re-maining files show the consensus trees generated for the designations of donor groups. These are the consensus topologies returned by the program MrBayes 3.2, showing in each node, its posterior prob-ability. Whenever a tree built with Phyml showed the same node, the bootstrap value was added man-ually. The bar shows the number of expected substitutions. Image files were generated and edited using the program Dendroscope. [file 3241027.f1.zip › EHI_096710_.png]

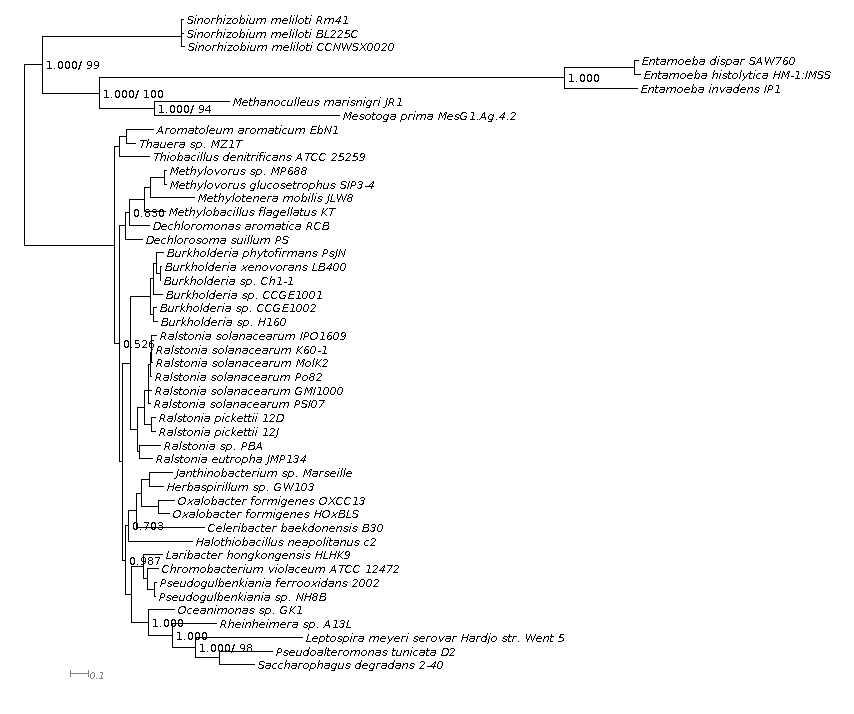

Supplement: Supplementary file 1 — The supplementary information consists of image files showing the consensus trees built for this research. Files are named with the AmoebaDB accession number of the horizontal gene transfer can-didate being tested. Files with the “_tre_ed.png” suffix are the trees built to evaluate the phylogenetic relevance of shorter alignments, these trees were built with the MrBayes 3.2 software. The posterior probability is shown for each node and the bar shows the expected number of substitutions. The re-maining files show the consensus trees generated for the designations of donor groups. These are the consensus topologies returned by the program MrBayes 3.2, showing in each node, its posterior prob-ability. Whenever a tree built with Phyml showed the same node, the bootstrap value was added man-ually. The bar shows the number of expected substitutions. Image files were generated and edited using the program Dendroscope. [file 3241027.f1.zip › EHI_098380_.png]

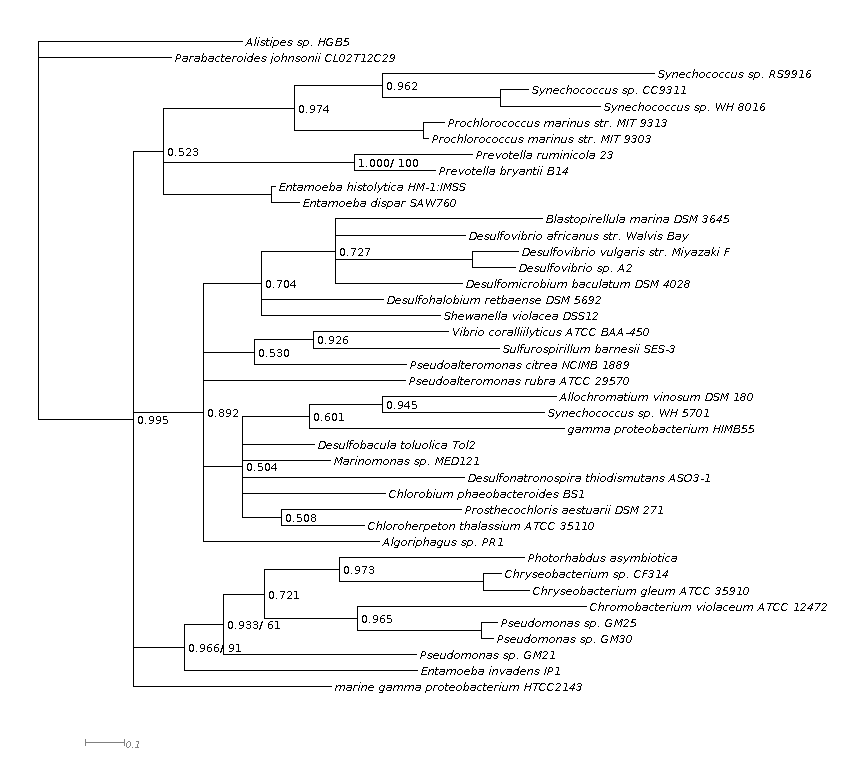

Supplement: Supplementary file 1 — The supplementary information consists of image files showing the consensus trees built for this research. Files are named with the AmoebaDB accession number of the horizontal gene transfer can-didate being tested. Files with the “_tre_ed.png” suffix are the trees built to evaluate the phylogenetic relevance of shorter alignments, these trees were built with the MrBayes 3.2 software. The posterior probability is shown for each node and the bar shows the expected number of substitutions. The re-maining files show the consensus trees generated for the designations of donor groups. These are the consensus topologies returned by the program MrBayes 3.2, showing in each node, its posterior prob-ability. Whenever a tree built with Phyml showed the same node, the bootstrap value was added man-ually. The bar shows the number of expected substitutions. Image files were generated and edited using the program Dendroscope. [file 3241027.f1.zip › EHI_100350_.png]

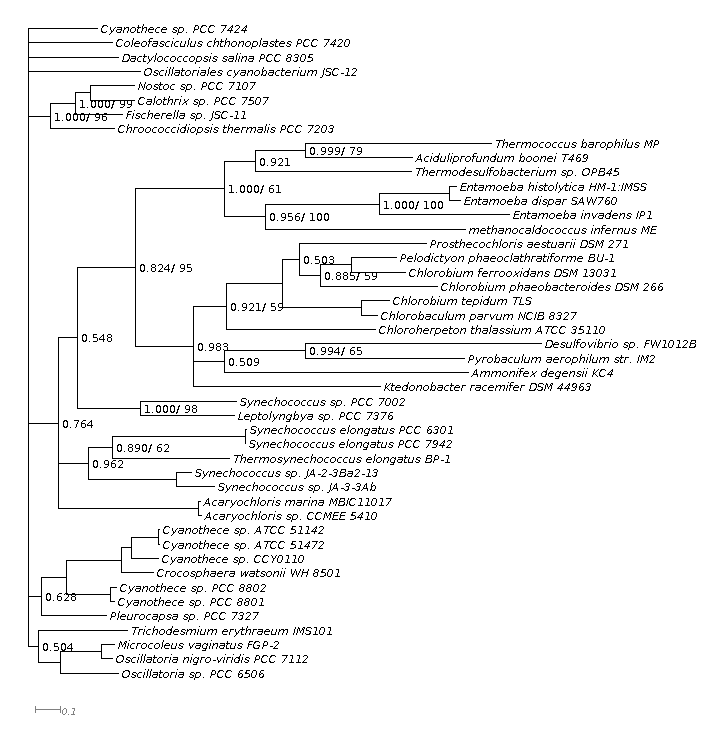

Supplement: Supplementary file 1 — The supplementary information consists of image files showing the consensus trees built for this research. Files are named with the AmoebaDB accession number of the horizontal gene transfer can-didate being tested. Files with the “_tre_ed.png” suffix are the trees built to evaluate the phylogenetic relevance of shorter alignments, these trees were built with the MrBayes 3.2 software. The posterior probability is shown for each node and the bar shows the expected number of substitutions. The re-maining files show the consensus trees generated for the designations of donor groups. These are the consensus topologies returned by the program MrBayes 3.2, showing in each node, its posterior prob-ability. Whenever a tree built with Phyml showed the same node, the bootstrap value was added man-ually. The bar shows the number of expected substitutions. Image files were generated and edited using the program Dendroscope. [file 3241027.f1.zip › EHI_100370_.png]

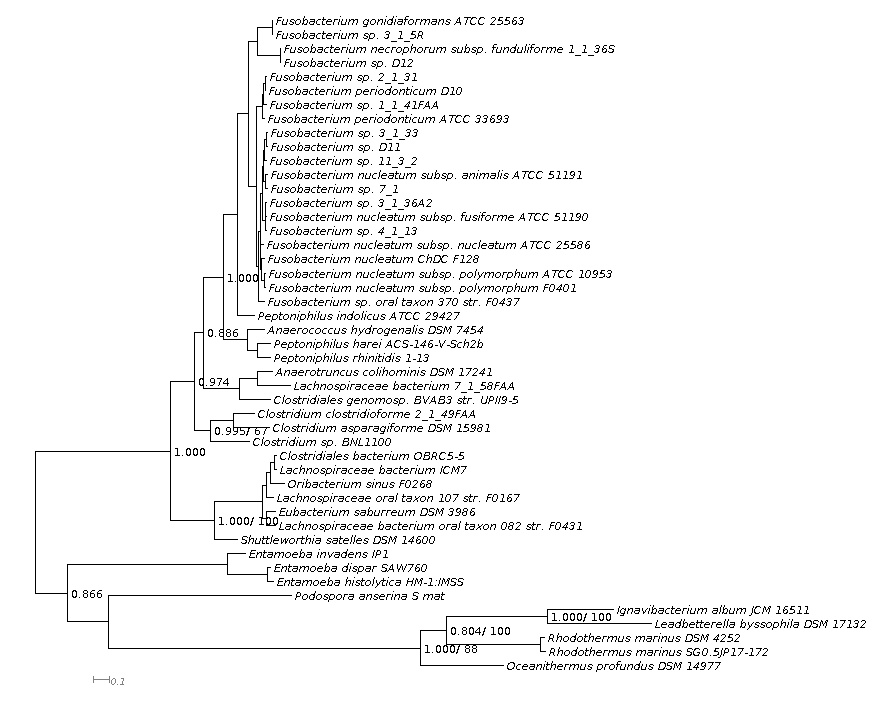

Supplement: Supplementary file 1 — The supplementary information consists of image files showing the consensus trees built for this research. Files are named with the AmoebaDB accession number of the horizontal gene transfer can-didate being tested. Files with the “_tre_ed.png” suffix are the trees built to evaluate the phylogenetic relevance of shorter alignments, these trees were built with the MrBayes 3.2 software. The posterior probability is shown for each node and the bar shows the expected number of substitutions. The re-maining files show the consensus trees generated for the designations of donor groups. These are the consensus topologies returned by the program MrBayes 3.2, showing in each node, its posterior prob-ability. Whenever a tree built with Phyml showed the same node, the bootstrap value was added man-ually. The bar shows the number of expected substitutions. Image files were generated and edited using the program Dendroscope. [file 3241027.f1.zip › EHI_111610_.png]

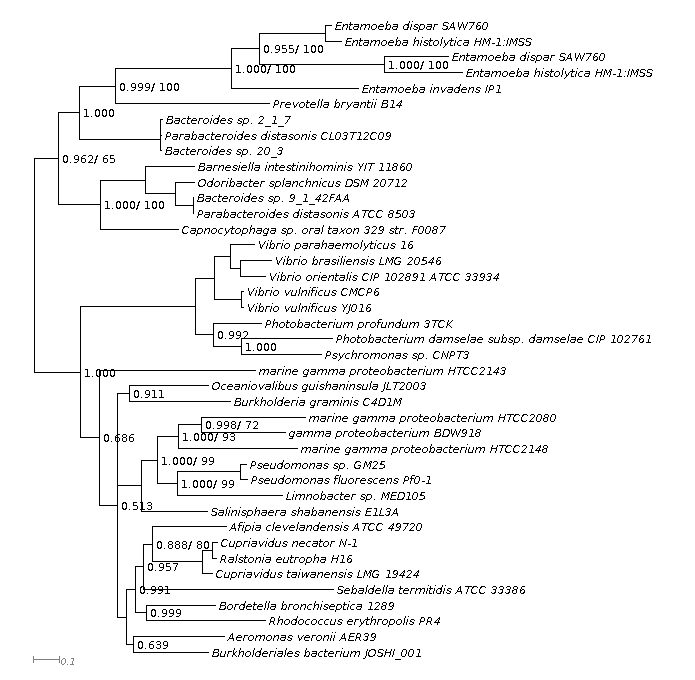

Supplement: Supplementary file 1 — The supplementary information consists of image files showing the consensus trees built for this research. Files are named with the AmoebaDB accession number of the horizontal gene transfer can-didate being tested. Files with the “_tre_ed.png” suffix are the trees built to evaluate the phylogenetic relevance of shorter alignments, these trees were built with the MrBayes 3.2 software. The posterior probability is shown for each node and the bar shows the expected number of substitutions. The re-maining files show the consensus trees generated for the designations of donor groups. These are the consensus topologies returned by the program MrBayes 3.2, showing in each node, its posterior prob-ability. Whenever a tree built with Phyml showed the same node, the bootstrap value was added man-ually. The bar shows the number of expected substitutions. Image files were generated and edited using the program Dendroscope. [file 3241027.f1.zip › EHI_115720_.png]

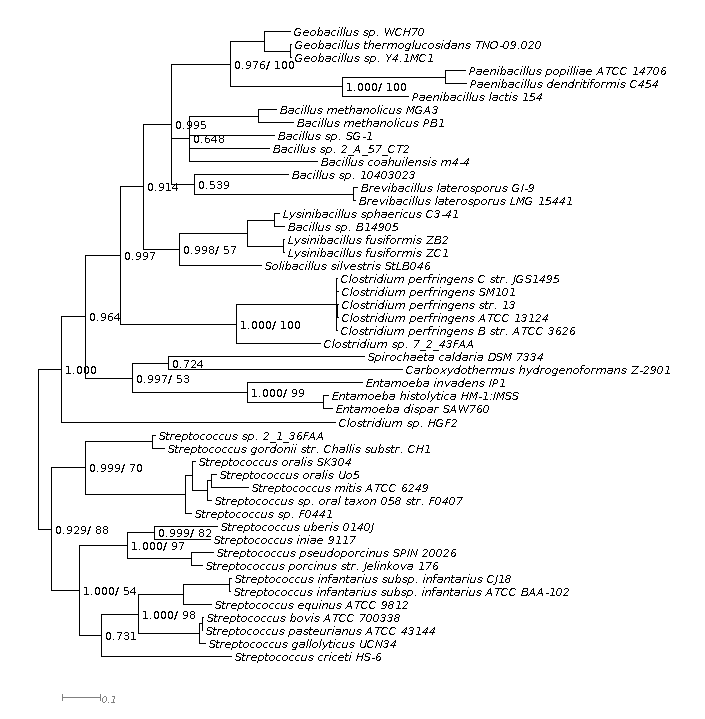

Supplement: Supplementary file 1 — The supplementary information consists of image files showing the consensus trees built for this research. Files are named with the AmoebaDB accession number of the horizontal gene transfer can-didate being tested. Files with the “_tre_ed.png” suffix are the trees built to evaluate the phylogenetic relevance of shorter alignments, these trees were built with the MrBayes 3.2 software. The posterior probability is shown for each node and the bar shows the expected number of substitutions. The re-maining files show the consensus trees generated for the designations of donor groups. These are the consensus topologies returned by the program MrBayes 3.2, showing in each node, its posterior prob-ability. Whenever a tree built with Phyml showed the same node, the bootstrap value was added man-ually. The bar shows the number of expected substitutions. Image files were generated and edited using the program Dendroscope. [file 3241027.f1.zip › EHI_121800_.png]

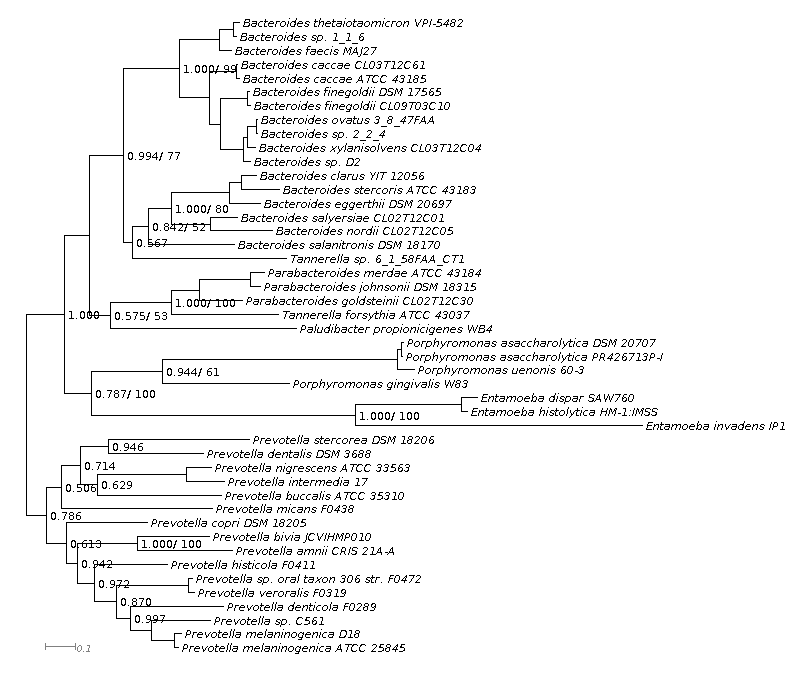

Supplement: Supplementary file 1 — The supplementary information consists of image files showing the consensus trees built for this research. Files are named with the AmoebaDB accession number of the horizontal gene transfer can-didate being tested. Files with the “_tre_ed.png” suffix are the trees built to evaluate the phylogenetic relevance of shorter alignments, these trees were built with the MrBayes 3.2 software. The posterior probability is shown for each node and the bar shows the expected number of substitutions. The re-maining files show the consensus trees generated for the designations of donor groups. These are the consensus topologies returned by the program MrBayes 3.2, showing in each node, its posterior prob-ability. Whenever a tree built with Phyml showed the same node, the bootstrap value was added man-ually. The bar shows the number of expected substitutions. Image files were generated and edited using the program Dendroscope. [file 3241027.f1.zip › EHI_123230_.png]

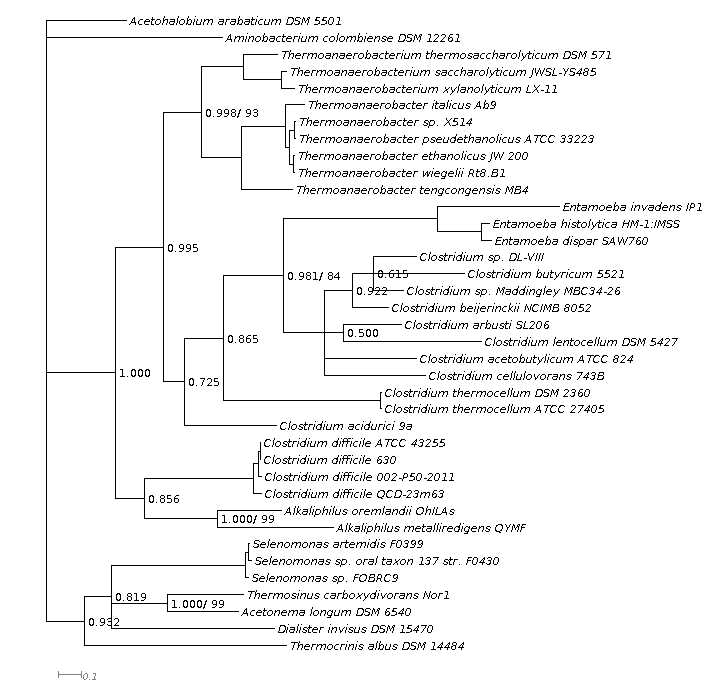

Supplement: Supplementary file 1 — The supplementary information consists of image files showing the consensus trees built for this research. Files are named with the AmoebaDB accession number of the horizontal gene transfer can-didate being tested. Files with the “_tre_ed.png” suffix are the trees built to evaluate the phylogenetic relevance of shorter alignments, these trees were built with the MrBayes 3.2 software. The posterior probability is shown for each node and the bar shows the expected number of substitutions. The re-maining files show the consensus trees generated for the designations of donor groups. These are the consensus topologies returned by the program MrBayes 3.2, showing in each node, its posterior prob-ability. Whenever a tree built with Phyml showed the same node, the bootstrap value was added man-ually. The bar shows the number of expected substitutions. Image files were generated and edited using the program Dendroscope. [file 3241027.f1.zip › EHI_129820_.png]

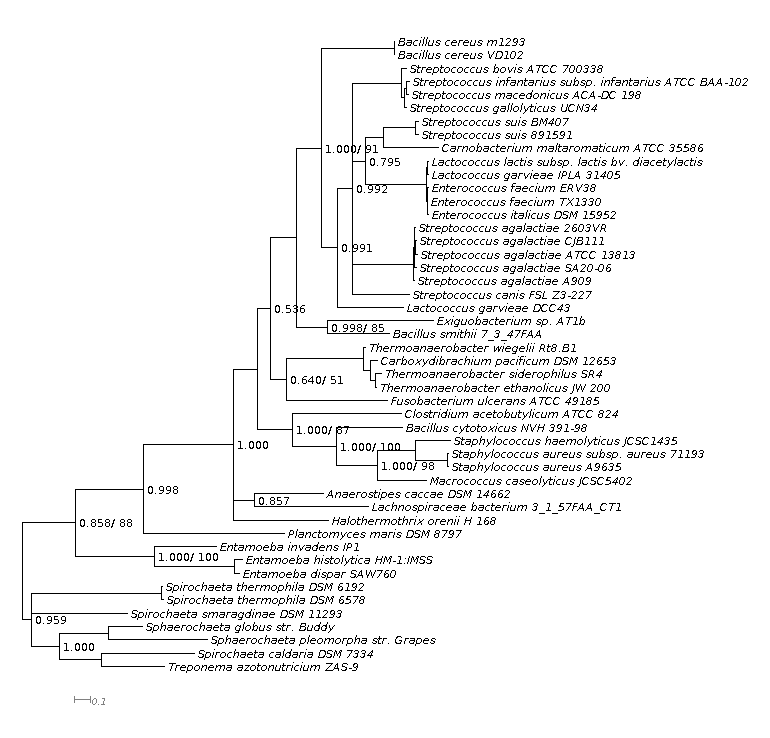

Supplement: Supplementary file 1 — The supplementary information consists of image files showing the consensus trees built for this research. Files are named with the AmoebaDB accession number of the horizontal gene transfer can-didate being tested. Files with the “_tre_ed.png” suffix are the trees built to evaluate the phylogenetic relevance of shorter alignments, these trees were built with the MrBayes 3.2 software. The posterior probability is shown for each node and the bar shows the expected number of substitutions. The re-maining files show the consensus trees generated for the designations of donor groups. These are the consensus topologies returned by the program MrBayes 3.2, showing in each node, its posterior prob-ability. Whenever a tree built with Phyml showed the same node, the bootstrap value was added man-ually. The bar shows the number of expected substitutions. Image files were generated and edited using the program Dendroscope. [file 3241027.f1.zip › EHI_138030_.png]

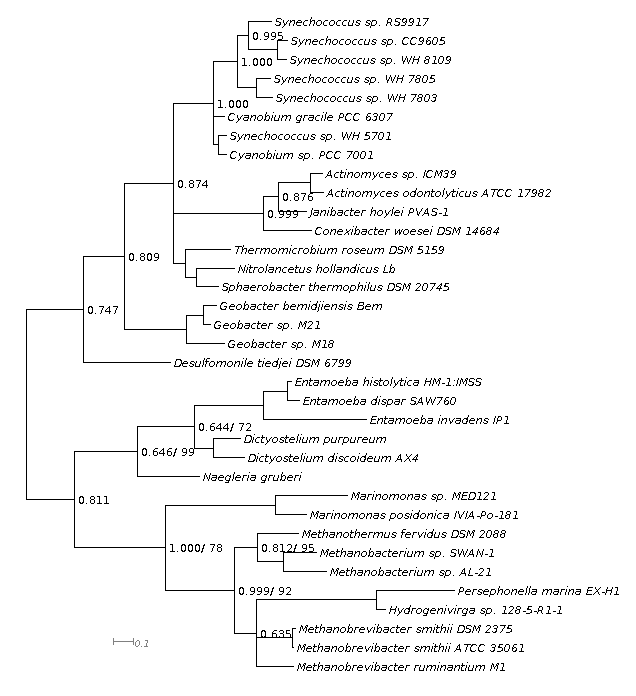

Supplement: Supplementary file 1 — The supplementary information consists of image files showing the consensus trees built for this research. Files are named with the AmoebaDB accession number of the horizontal gene transfer can-didate being tested. Files with the “_tre_ed.png” suffix are the trees built to evaluate the phylogenetic relevance of shorter alignments, these trees were built with the MrBayes 3.2 software. The posterior probability is shown for each node and the bar shows the expected number of substitutions. The re-maining files show the consensus trees generated for the designations of donor groups. These are the consensus topologies returned by the program MrBayes 3.2, showing in each node, its posterior prob-ability. Whenever a tree built with Phyml showed the same node, the bootstrap value was added man-ually. The bar shows the number of expected substitutions. Image files were generated and edited using the program Dendroscope. [file 3241027.f1.zip › EHI_140240_.png]

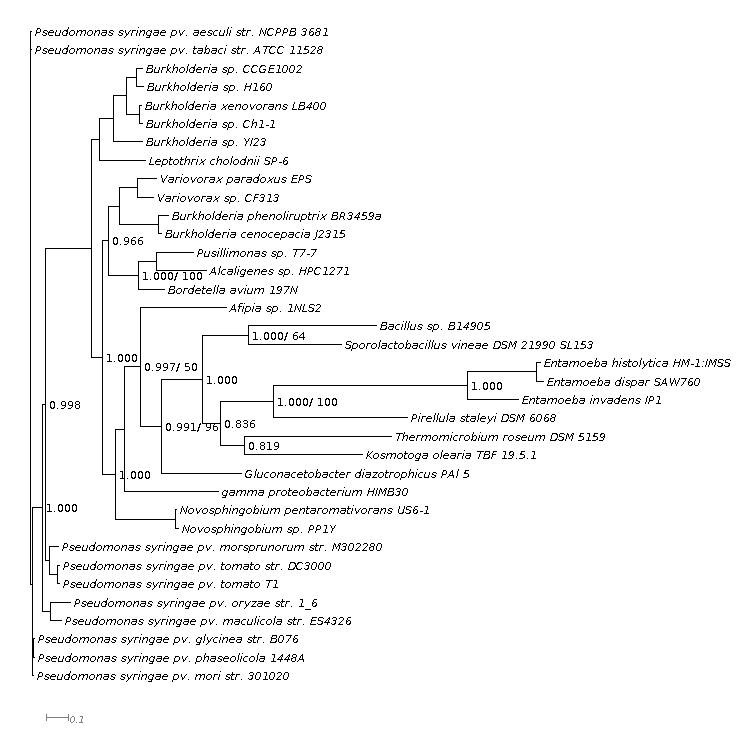

Supplement: Supplementary file 1 — The supplementary information consists of image files showing the consensus trees built for this research. Files are named with the AmoebaDB accession number of the horizontal gene transfer can-didate being tested. Files with the “_tre_ed.png” suffix are the trees built to evaluate the phylogenetic relevance of shorter alignments, these trees were built with the MrBayes 3.2 software. The posterior probability is shown for each node and the bar shows the expected number of substitutions. The re-maining files show the consensus trees generated for the designations of donor groups. These are the consensus topologies returned by the program MrBayes 3.2, showing in each node, its posterior prob-ability. Whenever a tree built with Phyml showed the same node, the bootstrap value was added man-ually. The bar shows the number of expected substitutions. Image files were generated and edited using the program Dendroscope. [file 3241027.f1.zip › EHI_143560_.png]

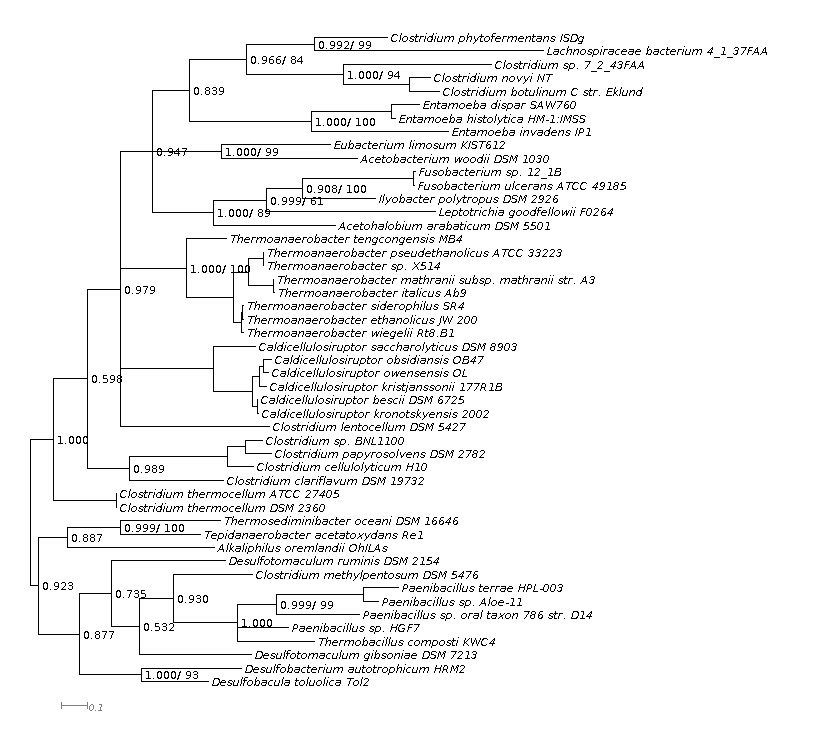

Supplement: Supplementary file 1 — The supplementary information consists of image files showing the consensus trees built for this research. Files are named with the AmoebaDB accession number of the horizontal gene transfer can-didate being tested. Files with the “_tre_ed.png” suffix are the trees built to evaluate the phylogenetic relevance of shorter alignments, these trees were built with the MrBayes 3.2 software. The posterior probability is shown for each node and the bar shows the expected number of substitutions. The re-maining files show the consensus trees generated for the designations of donor groups. These are the consensus topologies returned by the program MrBayes 3.2, showing in each node, its posterior prob-ability. Whenever a tree built with Phyml showed the same node, the bootstrap value was added man-ually. The bar shows the number of expected substitutions. Image files were generated and edited using the program Dendroscope. [file 3241027.f1.zip › EHI_148290_.png]

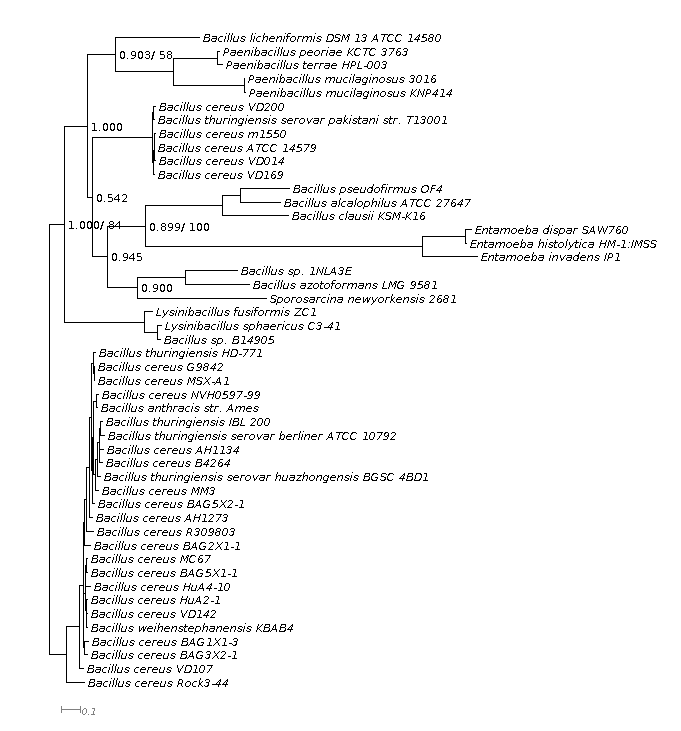

Supplement: Supplementary file 1 — The supplementary information consists of image files showing the consensus trees built for this research. Files are named with the AmoebaDB accession number of the horizontal gene transfer can-didate being tested. Files with the “_tre_ed.png” suffix are the trees built to evaluate the phylogenetic relevance of shorter alignments, these trees were built with the MrBayes 3.2 software. The posterior probability is shown for each node and the bar shows the expected number of substitutions. The re-maining files show the consensus trees generated for the designations of donor groups. These are the consensus topologies returned by the program MrBayes 3.2, showing in each node, its posterior prob-ability. Whenever a tree built with Phyml showed the same node, the bootstrap value was added man-ually. The bar shows the number of expected substitutions. Image files were generated and edited using the program Dendroscope. [file 3241027.f1.zip › EHI_150390_.png]

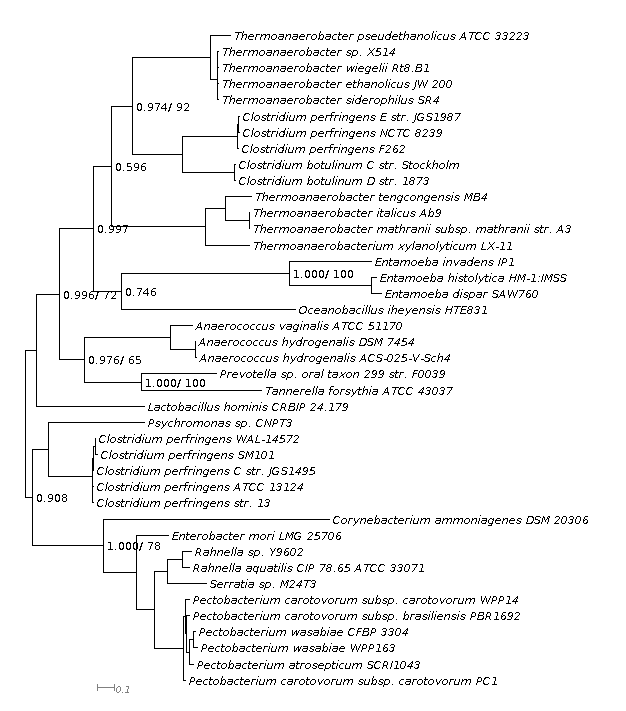

Supplement: Supplementary file 1 — The supplementary information consists of image files showing the consensus trees built for this research. Files are named with the AmoebaDB accession number of the horizontal gene transfer can-didate being tested. Files with the “_tre_ed.png” suffix are the trees built to evaluate the phylogenetic relevance of shorter alignments, these trees were built with the MrBayes 3.2 software. The posterior probability is shown for each node and the bar shows the expected number of substitutions. The re-maining files show the consensus trees generated for the designations of donor groups. These are the consensus topologies returned by the program MrBayes 3.2, showing in each node, its posterior prob-ability. Whenever a tree built with Phyml showed the same node, the bootstrap value was added man-ually. The bar shows the number of expected substitutions. Image files were generated and edited using the program Dendroscope. [file 3241027.f1.zip › EHI_151930_.png]

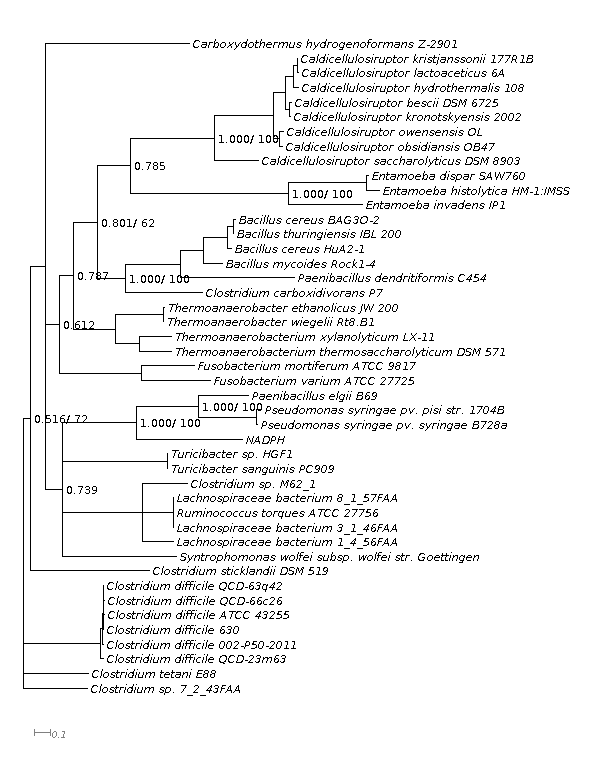

Supplement: Supplementary file 1 — The supplementary information consists of image files showing the consensus trees built for this research. Files are named with the AmoebaDB accession number of the horizontal gene transfer can-didate being tested. Files with the “_tre_ed.png” suffix are the trees built to evaluate the phylogenetic relevance of shorter alignments, these trees were built with the MrBayes 3.2 software. The posterior probability is shown for each node and the bar shows the expected number of substitutions. The re-maining files show the consensus trees generated for the designations of donor groups. These are the consensus topologies returned by the program MrBayes 3.2, showing in each node, its posterior prob-ability. Whenever a tree built with Phyml showed the same node, the bootstrap value was added man-ually. The bar shows the number of expected substitutions. Image files were generated and edited using the program Dendroscope. [file 3241027.f1.zip › EHI_153000_.png]

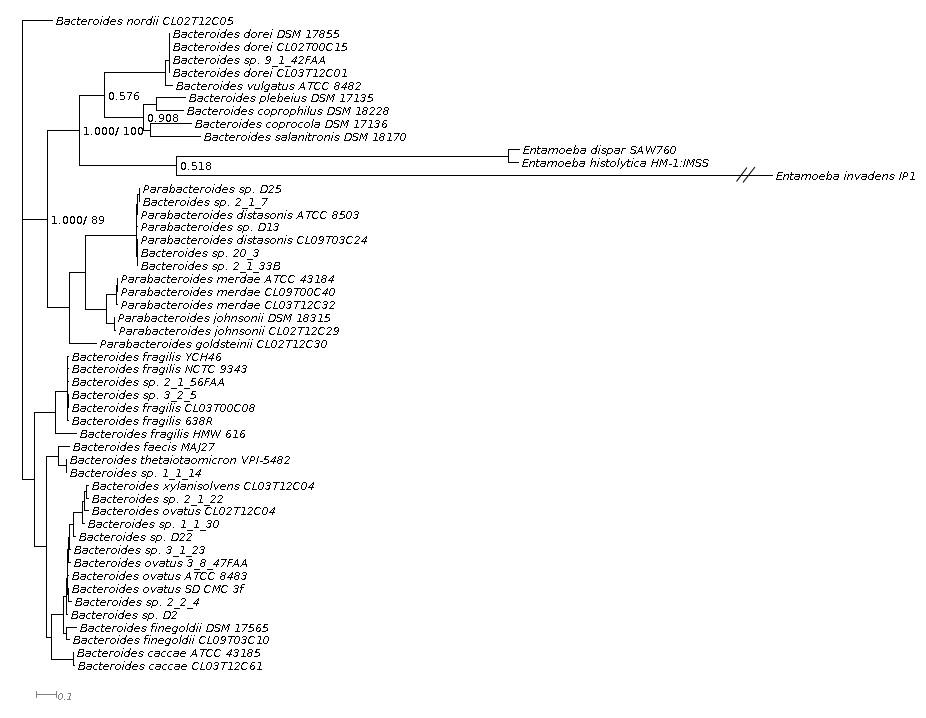

Supplement: Supplementary file 1 — The supplementary information consists of image files showing the consensus trees built for this research. Files are named with the AmoebaDB accession number of the horizontal gene transfer can-didate being tested. Files with the “_tre_ed.png” suffix are the trees built to evaluate the phylogenetic relevance of shorter alignments, these trees were built with the MrBayes 3.2 software. The posterior probability is shown for each node and the bar shows the expected number of substitutions. The re-maining files show the consensus trees generated for the designations of donor groups. These are the consensus topologies returned by the program MrBayes 3.2, showing in each node, its posterior prob-ability. Whenever a tree built with Phyml showed the same node, the bootstrap value was added man-ually. The bar shows the number of expected substitutions. Image files were generated and edited using the program Dendroscope. [file 3241027.f1.zip › EHI_153350_.png]

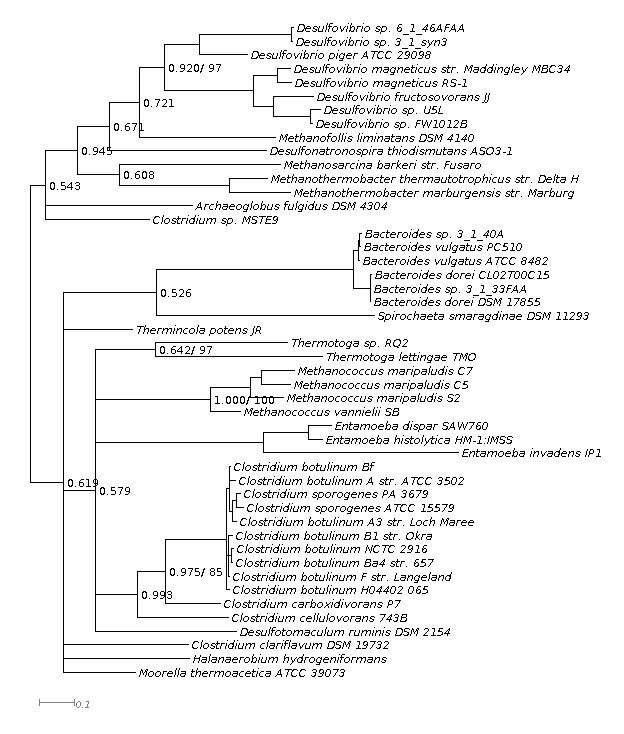

Supplement: Supplementary file 1 — The supplementary information consists of image files showing the consensus trees built for this research. Files are named with the AmoebaDB accession number of the horizontal gene transfer can-didate being tested. Files with the “_tre_ed.png” suffix are the trees built to evaluate the phylogenetic relevance of shorter alignments, these trees were built with the MrBayes 3.2 software. The posterior probability is shown for each node and the bar shows the expected number of substitutions. The re-maining files show the consensus trees generated for the designations of donor groups. These are the consensus topologies returned by the program MrBayes 3.2, showing in each node, its posterior prob-ability. Whenever a tree built with Phyml showed the same node, the bootstrap value was added man-ually. The bar shows the number of expected substitutions. Image files were generated and edited using the program Dendroscope. [file 3241027.f1.zip › EHI_153410_.png]

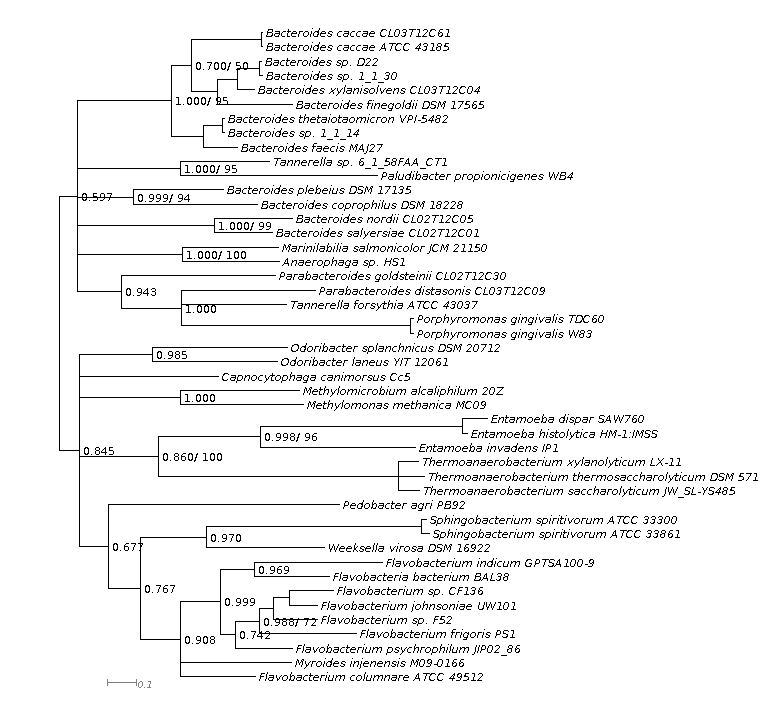

Supplement: Supplementary file 1 — The supplementary information consists of image files showing the consensus trees built for this research. Files are named with the AmoebaDB accession number of the horizontal gene transfer can-didate being tested. Files with the “_tre_ed.png” suffix are the trees built to evaluate the phylogenetic relevance of shorter alignments, these trees were built with the MrBayes 3.2 software. The posterior probability is shown for each node and the bar shows the expected number of substitutions. The re-maining files show the consensus trees generated for the designations of donor groups. These are the consensus topologies returned by the program MrBayes 3.2, showing in each node, its posterior prob-ability. Whenever a tree built with Phyml showed the same node, the bootstrap value was added man-ually. The bar shows the number of expected substitutions. Image files were generated and edited using the program Dendroscope. [file 3241027.f1.zip › EHI_153470_.png]

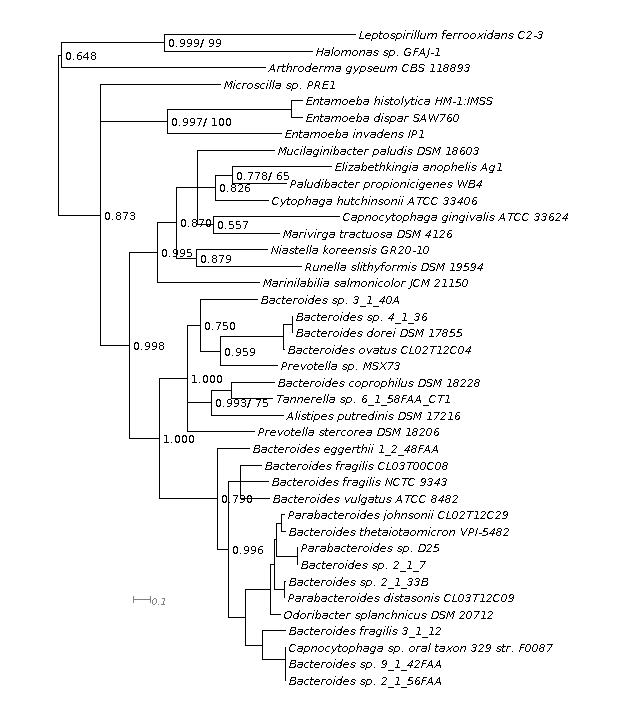

Supplement: Supplementary file 1 — The supplementary information consists of image files showing the consensus trees built for this research. Files are named with the AmoebaDB accession number of the horizontal gene transfer can-didate being tested. Files with the “_tre_ed.png” suffix are the trees built to evaluate the phylogenetic relevance of shorter alignments, these trees were built with the MrBayes 3.2 software. The posterior probability is shown for each node and the bar shows the expected number of substitutions. The re-maining files show the consensus trees generated for the designations of donor groups. These are the consensus topologies returned by the program MrBayes 3.2, showing in each node, its posterior prob-ability. Whenever a tree built with Phyml showed the same node, the bootstrap value was added man-ually. The bar shows the number of expected substitutions. Image files were generated and edited using the program Dendroscope. [file 3241027.f1.zip › EHI_161000_.png]

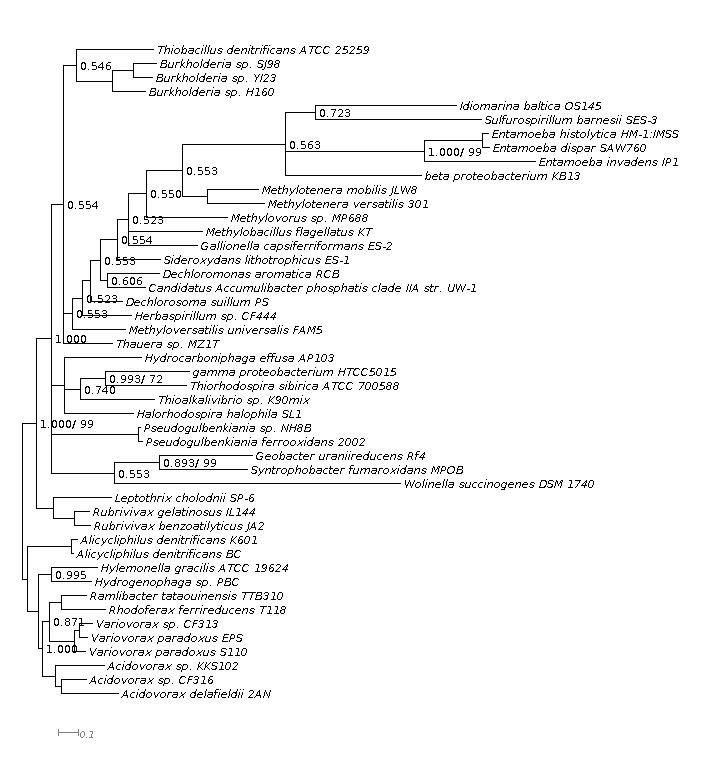

Supplement: Supplementary file 1 — The supplementary information consists of image files showing the consensus trees built for this research. Files are named with the AmoebaDB accession number of the horizontal gene transfer can-didate being tested. Files with the “_tre_ed.png” suffix are the trees built to evaluate the phylogenetic relevance of shorter alignments, these trees were built with the MrBayes 3.2 software. The posterior probability is shown for each node and the bar shows the expected number of substitutions. The re-maining files show the consensus trees generated for the designations of donor groups. These are the consensus topologies returned by the program MrBayes 3.2, showing in each node, its posterior prob-ability. Whenever a tree built with Phyml showed the same node, the bootstrap value was added man-ually. The bar shows the number of expected substitutions. Image files were generated and edited using the program Dendroscope. [file 3241027.f1.zip › EHI_169860_.png]

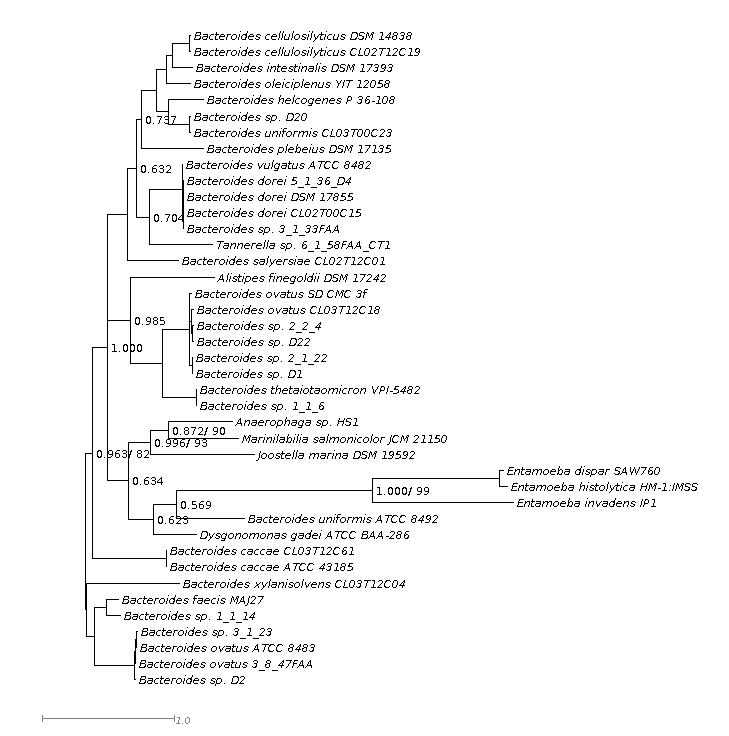

Supplement: Supplementary file 1 — The supplementary information consists of image files showing the consensus trees built for this research. Files are named with the AmoebaDB accession number of the horizontal gene transfer can-didate being tested. Files with the “_tre_ed.png” suffix are the trees built to evaluate the phylogenetic relevance of shorter alignments, these trees were built with the MrBayes 3.2 software. The posterior probability is shown for each node and the bar shows the expected number of substitutions. The re-maining files show the consensus trees generated for the designations of donor groups. These are the consensus topologies returned by the program MrBayes 3.2, showing in each node, its posterior prob-ability. Whenever a tree built with Phyml showed the same node, the bootstrap value was added man-ually. The bar shows the number of expected substitutions. Image files were generated and edited using the program Dendroscope. [file 3241027.f1.zip › EHI_170020_.png]

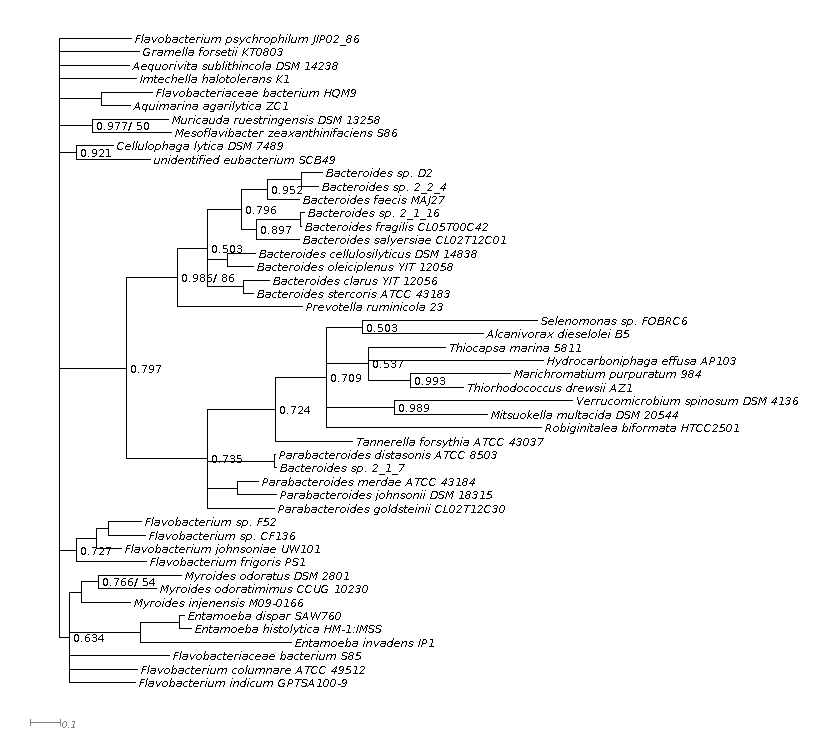

Supplement: Supplementary file 1 — The supplementary information consists of image files showing the consensus trees built for this research. Files are named with the AmoebaDB accession number of the horizontal gene transfer can-didate being tested. Files with the “_tre_ed.png” suffix are the trees built to evaluate the phylogenetic relevance of shorter alignments, these trees were built with the MrBayes 3.2 software. The posterior probability is shown for each node and the bar shows the expected number of substitutions. The re-maining files show the consensus trees generated for the designations of donor groups. These are the consensus topologies returned by the program MrBayes 3.2, showing in each node, its posterior prob-ability. Whenever a tree built with Phyml showed the same node, the bootstrap value was added man-ually. The bar shows the number of expected substitutions. Image files were generated and edited using the program Dendroscope. [file 3241027.f1.zip › EHI_178070_.png]

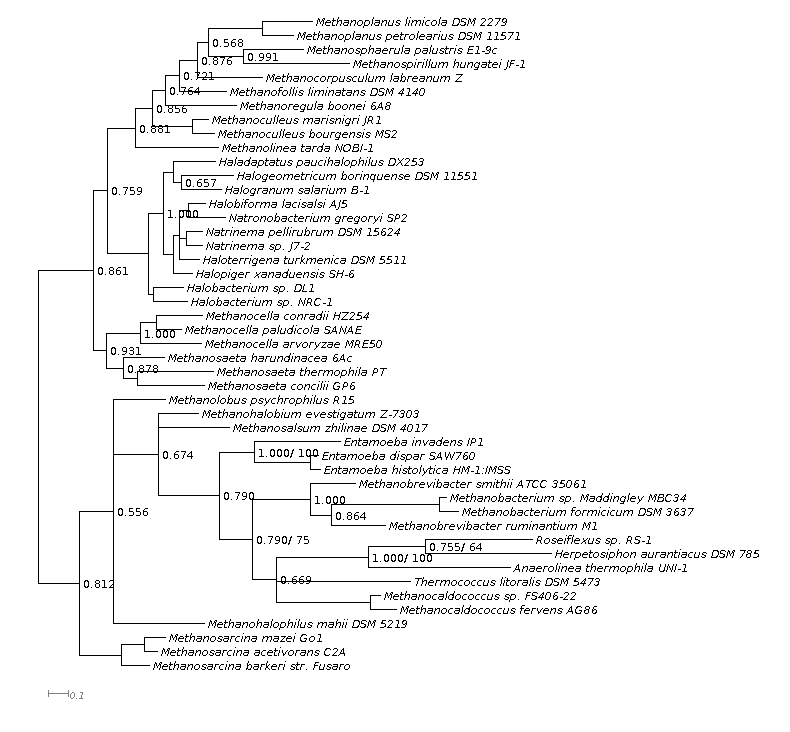

Supplement: Supplementary file 1 — The supplementary information consists of image files showing the consensus trees built for this research. Files are named with the AmoebaDB accession number of the horizontal gene transfer can-didate being tested. Files with the “_tre_ed.png” suffix are the trees built to evaluate the phylogenetic relevance of shorter alignments, these trees were built with the MrBayes 3.2 software. The posterior probability is shown for each node and the bar shows the expected number of substitutions. The re-maining files show the consensus trees generated for the designations of donor groups. These are the consensus topologies returned by the program MrBayes 3.2, showing in each node, its posterior prob-ability. Whenever a tree built with Phyml showed the same node, the bootstrap value was added man-ually. The bar shows the number of expected substitutions. Image files were generated and edited using the program Dendroscope. [file 3241027.f1.zip › EHI_178490_.png]

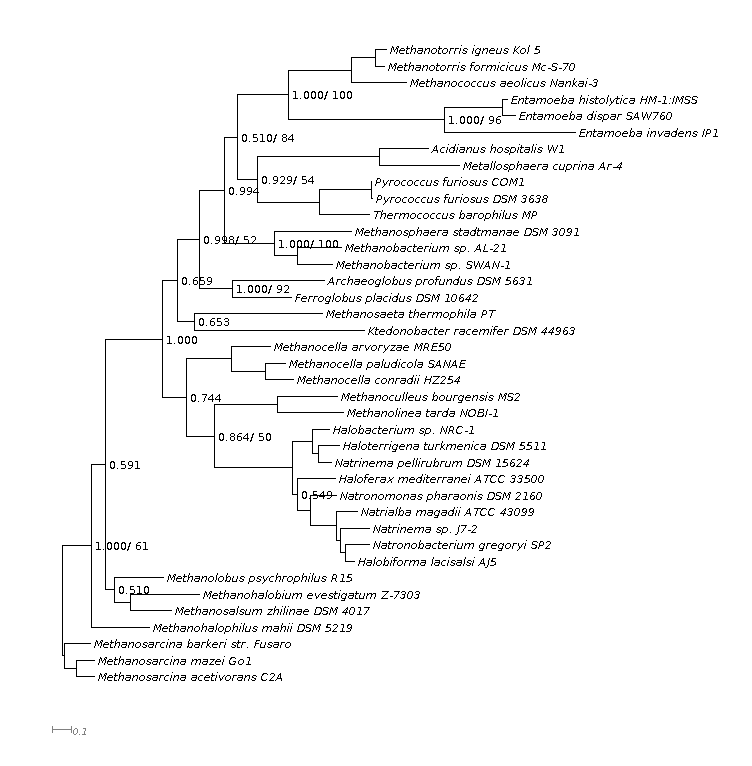

Supplement: Supplementary file 1 — The supplementary information consists of image files showing the consensus trees built for this research. Files are named with the AmoebaDB accession number of the horizontal gene transfer can-didate being tested. Files with the “_tre_ed.png” suffix are the trees built to evaluate the phylogenetic relevance of shorter alignments, these trees were built with the MrBayes 3.2 software. The posterior probability is shown for each node and the bar shows the expected number of substitutions. The re-maining files show the consensus trees generated for the designations of donor groups. These are the consensus topologies returned by the program MrBayes 3.2, showing in each node, its posterior prob-ability. Whenever a tree built with Phyml showed the same node, the bootstrap value was added man-ually. The bar shows the number of expected substitutions. Image files were generated and edited using the program Dendroscope. [file 3241027.f1.zip › EHI_194410_.png]

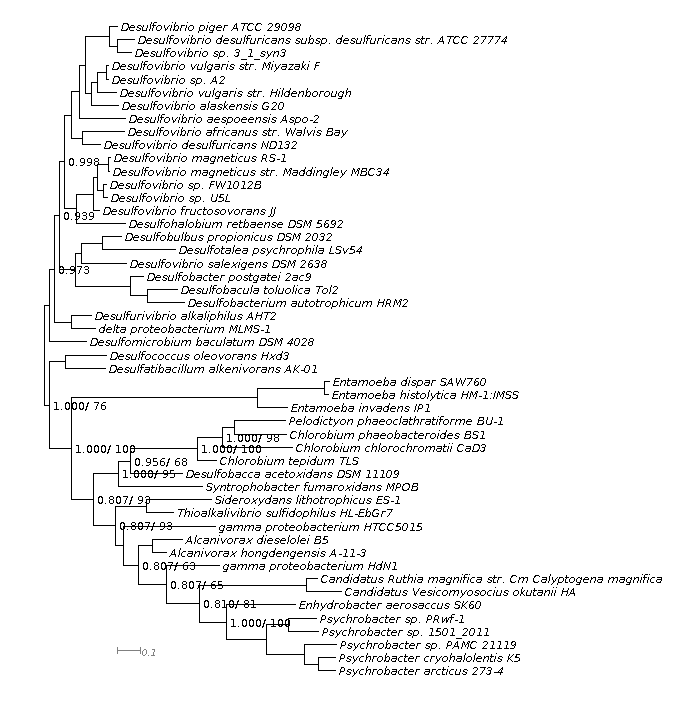

Supplement: Supplementary file 1 — The supplementary information consists of image files showing the consensus trees built for this research. Files are named with the AmoebaDB accession number of the horizontal gene transfer can-didate being tested. Files with the “_tre_ed.png” suffix are the trees built to evaluate the phylogenetic relevance of shorter alignments, these trees were built with the MrBayes 3.2 software. The posterior probability is shown for each node and the bar shows the expected number of substitutions. The re-maining files show the consensus trees generated for the designations of donor groups. These are the consensus topologies returned by the program MrBayes 3.2, showing in each node, its posterior prob-ability. Whenever a tree built with Phyml showed the same node, the bootstrap value was added man-ually. The bar shows the number of expected substitutions. Image files were generated and edited using the program Dendroscope. [file 3241027.f1.zip › EHI_197160_.png]

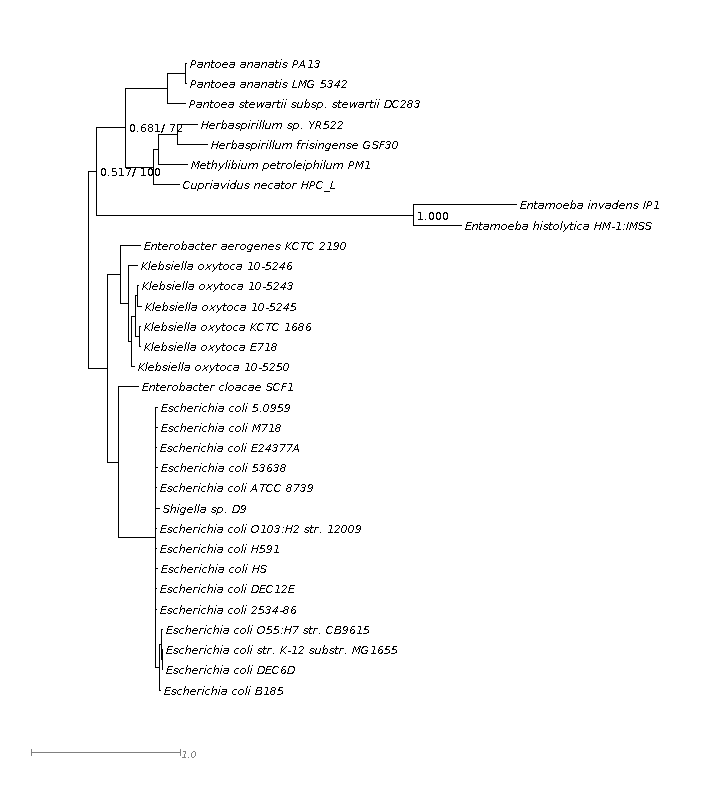

Supplement: Supplementary file 1 — The supplementary information consists of image files showing the consensus trees built for this research. Files are named with the AmoebaDB accession number of the horizontal gene transfer can-didate being tested. Files with the “_tre_ed.png” suffix are the trees built to evaluate the phylogenetic relevance of shorter alignments, these trees were built with the MrBayes 3.2 software. The posterior probability is shown for each node and the bar shows the expected number of substitutions. The re-maining files show the consensus trees generated for the designations of donor groups. These are the consensus topologies returned by the program MrBayes 3.2, showing in each node, its posterior prob-ability. Whenever a tree built with Phyml showed the same node, the bootstrap value was added man-ually. The bar shows the number of expected substitutions. Image files were generated and edited using the program Dendroscope. [file 3241027.f1.zip › EHI_198610_.png]

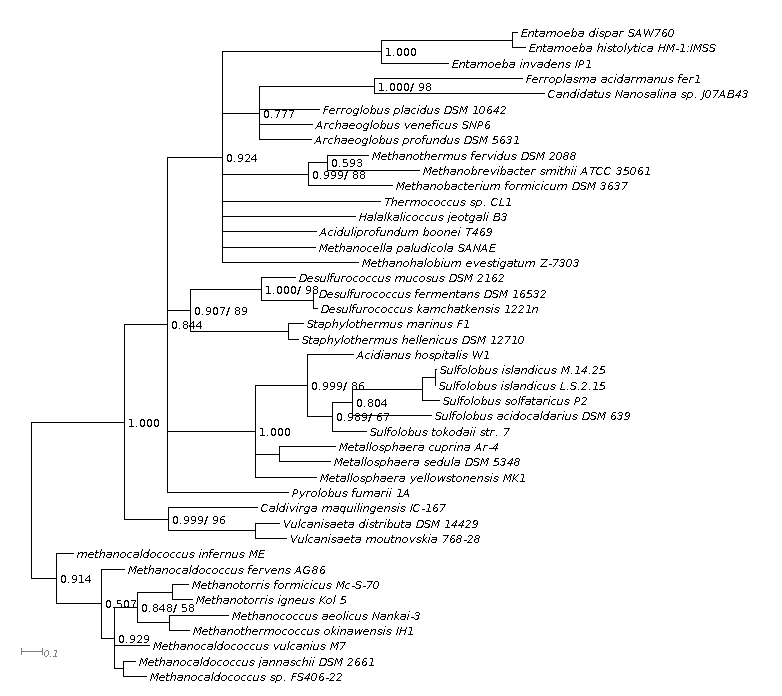

Supplement: Supplementary file 1 — The supplementary information consists of image files showing the consensus trees built for this research. Files are named with the AmoebaDB accession number of the horizontal gene transfer can-didate being tested. Files with the “_tre_ed.png” suffix are the trees built to evaluate the phylogenetic relevance of shorter alignments, these trees were built with the MrBayes 3.2 software. The posterior probability is shown for each node and the bar shows the expected number of substitutions. The re-maining files show the consensus trees generated for the designations of donor groups. These are the consensus topologies returned by the program MrBayes 3.2, showing in each node, its posterior prob-ability. Whenever a tree built with Phyml showed the same node, the bootstrap value was added man-ually. The bar shows the number of expected substitutions. Image files were generated and edited using the program Dendroscope. [file 3241027.f1.zip › EHI_198800_.png]

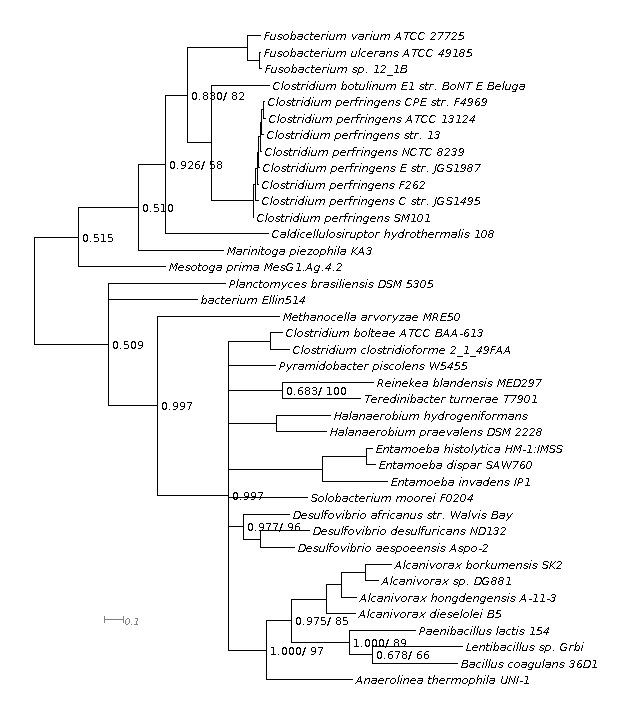

Supplement: Supplementary file 1 — The supplementary information consists of image files showing the consensus trees built for this research. Files are named with the AmoebaDB accession number of the horizontal gene transfer can-didate being tested. Files with the “_tre_ed.png” suffix are the trees built to evaluate the phylogenetic relevance of shorter alignments, these trees were built with the MrBayes 3.2 software. The posterior probability is shown for each node and the bar shows the expected number of substitutions. The re-maining files show the consensus trees generated for the designations of donor groups. These are the consensus topologies returned by the program MrBayes 3.2, showing in each node, its posterior prob-ability. Whenever a tree built with Phyml showed the same node, the bootstrap value was added man-ually. The bar shows the number of expected substitutions. Image files were generated and edited using the program Dendroscope. [file 3241027.f1.zip › EHI_199080_.png]

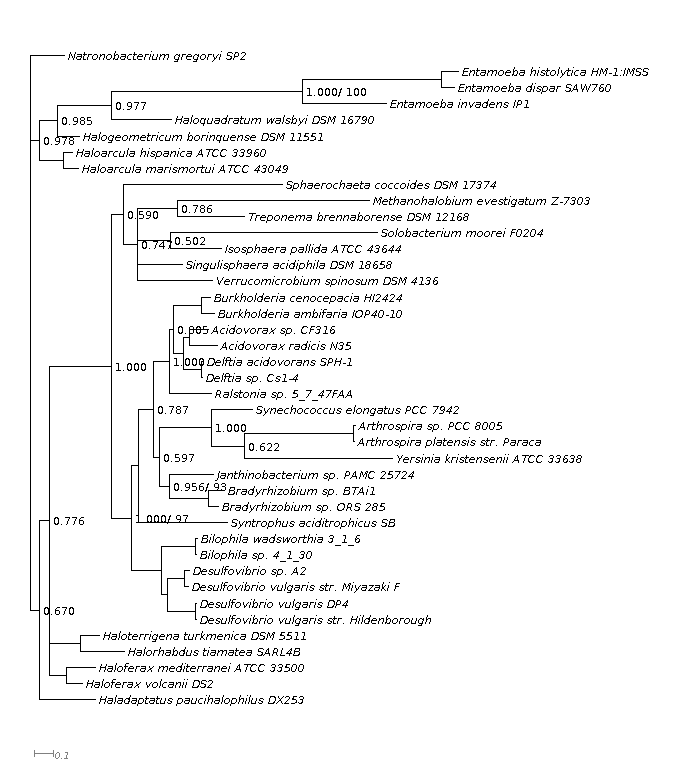

Supplement: Supplementary file 1 — The supplementary information consists of image files showing the consensus trees built for this research. Files are named with the AmoebaDB accession number of the horizontal gene transfer can-didate being tested. Files with the “_tre_ed.png” suffix are the trees built to evaluate the phylogenetic relevance of shorter alignments, these trees were built with the MrBayes 3.2 software. The posterior probability is shown for each node and the bar shows the expected number of substitutions. The re-maining files show the consensus trees generated for the designations of donor groups. These are the consensus topologies returned by the program MrBayes 3.2, showing in each node, its posterior prob-ability. Whenever a tree built with Phyml showed the same node, the bootstrap value was added man-ually. The bar shows the number of expected substitutions. Image files were generated and edited using the program Dendroscope. [file 3241027.f1.zip › EHI_202040_.png]
